# Metformin sensitizes esophageal squamous cell carcinoma to Vγ9Vδ2 T cell-mediated cytotoxicity by upregulating BTN3A1 and BTN2A1

**DOI:** 10.1038/s41419-026-08924-6

**Published:** 2026-06-06

**Authors:** Zishan Yang, Yadi Liu, Yixuan Han, Lijun Tan, Suli Wang, Shenglan Zhang, Chenyang Li, Piaoyi He, Yaxin Zhang, Yilong Ji, Zhinan Yin, Jian Li, Feng Ren

**Affiliations:** 1 Xinxiang Key Laboratory of Tumor Vaccine and Immunotherapy, School of Basic Medical Sciences, Henan Medical University, Xinxiang, Henan PR China; 2 Henan Research Center for Engineering Technology in Digestive Tract Tumor Immune Digital Decoding and Cell Therapy, Henan Medical University, Xinxiang, Henan PR China; 3 Henan International Joint Laboratory of Immunity and Targeted Therapy for Liver-Intestinal Tumors, Henan Medical University, Xinxiang, Henan PR China; 4 The Biomedical Translational Research Institute, Faculty of Medical Science, Jinan University, Guangzhou, Guangdong PR China; 5 Department of Pathology, The First Affiliated Hospital of Henan Medical University, Xinxiang, Henan China

**Keywords:** Immunization, Innate lymphoid cells

## Abstract

Metformin, a first-line anti-diabetic agent, exhibits broad-spectrum antitumor properties, though its underlying immunomodulatory mechanisms remain incompletely characterized. Here, we demonstrate that metformin significantly upregulates BTN3A1 and BTN2A1 expression on esophageal cancer cells in an AMPK-dependent manner, thereby sensitizing them to Vγ9Vδ2 T cell-mediated cytotoxicity. This molecular priming enhanced tumor immunogenicity, leading to synergistic tumor cell killing in vitro and potent suppression of tumor growth in xenograft models. Mechanistically, metformin-induced BTN3A1/BTN2A1 upregulation promoted Vγ9Vδ2 T cell activation, and Granzyme B-mediated apoptosis in tumor tissues. The combination therapy demonstrated excellent tolerability without observable systemic toxicity. Moreover, Integrating GEPIA3 database and clinical specimen analyses, we find that BTN3A1 and BTN2A1 are highly but heterogeneously expressed in esophageal cancer tissues, and that metformin‑mediated upregulation may restore sensitivity to Vγ9Vδ2 T cell immunotherapy particularly in patients with low baseline expression—uncovering a novel immunomodulatory function of metformin that provides a compelling rationale for its repurposing as a combinatorial agent against immunologically cold tumors such as esophageal carcinoma.

## Introduction

Esophageal cancer (EC), primarily comprising esophageal squamous cell carcinoma (ESCC) and esophageal adenocarcinoma (EAC), is a highly aggressive disease with a dismal prognosis. ESCC is the dominant subtype globally and accounts for over 90% of cases in high-risk regions like China, while EAC is more common in Western countries [1, 2]. The global burden is severe, with approximately 511,000 new cases and 445,000 deaths annually (2022 data), making it the 11th most commonly diagnosed cancer and the 7th leading cause of cancer death worldwide [3, 4]. China bears a disproportionate share, accounting for nearly half of the global cases and deaths [5]. Current standard treatments, including surgery, chemoradiotherapy, and increasingly, immune checkpoint inhibitors (ICIs) combined with chemotherapy, have improved outcomes but face significant limitations: therapeutic responses are highly variable among individuals, many patients develop resistance, and the overall 5-year survival rate remains low at approximately 22% [6, 7]. There is an urgent need to explore novel immune cell-based therapies or rational combination strategies to enhance treatment efficacy and overcome resistance in this challenging malignancy.

In this context, γδ T cells, specifically the Vγ9Vδ2 subset, which is the dominate subset among γδ T cells in human peripheral blood, represent a promising frontier in cancer immunotherapy due to their unique MHC-independent recognition of tumors [8]. Unlike αβ T cells, they do not require peptide presentation by MHC molecules, enabling them to target a broad spectrum of cancers—including those with low mutational burden or MHC downregulation [9]. A central mechanism involves the recognition of phosphoantigens (e.g., IPP) through the butyrophilin complex, notably BTN3A1 and BTN2A1. These molecules mediate “inside-out” signaling, where intracellular phosphoantigen accumulation induces conformational changes in BTN3A1, facilitating direct or indirect activation of Vγ9Vδ2 TCRs [10, 11]. This mechanism allows γδ T cells to sense metabolic dysregulation in transformed cells early in oncogenesis [12]. Additionally, building upon our group’s prior clinical studies, which demonstrated the safety and preliminary efficacy of allogeneic Vγ9Vδ2 T-cell therapy in cohorts with cholangiocarcinoma, advanced pancreatic cancer, and late-stage lung or liver cancer, as well as its role in promoting pulmonary lesion repair in a case report [13–16], we further highlight their inherent suitability for “off-the-shelf” therapies. Their capacity for allogeneic use without provoking severe graft-versus-host disease, combined with innate-like cytotoxicity and adaptive memory functions, positions them as versatile effector cells.

Despite their potential, γδ T cell-based therapies face substantial hurdles. The functional duality of γδ T cells is a major concern: certain subsets, particularly IL-17 producers, can promote tumor progression via angiogenesis and immunosuppression [17]. Moreover, clinical trials using expanded polyclonal γδ T cells have shown limited efficacy, often due to poor persistence, exhaustion, and tumor microenvironment-driven suppression [18, 19]. The reliance on BTN3A1/BTN2A1- mediated activation, while a strength, also presents a limitation, as tumors may downregulate these molecules or alter phosphoantigen metabolism to evade detection [20, 21]. The extreme diversity of γδTCRs and the lack of conserved ligands further complicate therapeutic targeting and preclinical modeling. Given these constraints, there is an urgent need to explore novel combination strategies, such as integrating γδ T cell therapies with immune checkpoint inhibitors, bispecific engagers, or CAR engineering, to enhance persistence, overcome tumor immune evasion, and unlock their full therapeutic potential.

Intriguingly, the widely used anti-diabetic drug metformin presents a novel solution to this challenge. Epidemiological studies reveal that metformin use is associated with a significantly reduced risk of developing ESCC and improved overall survival in patients, showing a dose-dependent relationship [22, 23]. Its antitumor mechanisms are multifaceted. Metformin directly induces tumor cell apoptosis and protective autophagy by inhibiting the Stat3-Bcl-2 pathway [24], while also suppressing radiation-induced invasion and epithelial-mesenchymal transition (EMT) via blockade of TGF-β/Smad signaling [25]. Furthermore, it reprograms the immunosuppressive tumor microenvironment by enhancing the infiltration and function of cytotoxic CD8^+^ T cells and M1 macrophages, while reducing myeloid-derived suppressor cells (MDSCs) [26]. Notably, a key immunomodulatory mechanism involves the upregulation of butyrophilin family members BTN3A1 and BTN2A1, which are critical for activating Vγ9Vδ2 T cells, a major subset of γδ T cells with potent anti-tumor cytotoxicity [27, 28]. This specific action, alongside its established metabolic effects through AMPK activation and mTOR inhibition, positions metformin as a promising immunometabolic adjuvant [29–31]. Targeting the BTN3A1/BTN2A1 axis thus provides a novel strategic rationale for developing new immunotherapies or combination regimens in esophageal cancer.

In this study, we demonstrate that the combination of metformin with Vγ9Vδ2 T cells exerts a potent synergistic antitumor effect against esophageal squamous cell carcinoma in vivo. This enhanced therapeutic efficacy is associated with significantly increased tumor cell apoptosis and augmented Vγ9Vδ2 T cell cytotoxicity within tumor tissues. Mechanistically, we reveal that metformin substantially upregulates the expression of BTN3A1 and BTN2A1 on tumor cells, thereby sensitizing them to Vγ9Vδ2 T cell-mediated killing. The specificity of this mechanism is confirmed by the finding that BTN3A1 blockade effectively abrogates the enhanced cytotoxicity. Our work elucidates a previously unrecognized immunomodulatory mechanism of metformin and provides a compelling rationale for repurposing this clinically approved drug as a combinatorial agent to potentiate γδ T cell-based immunotherapy.

## Materials and methods

### Human blood specimen

Peripheral blood samples were collected from 40 healthy volunteers. All blood samples were processed immediately for the isolation of peripheral blood mononuclear cells (PBMCs), which were subsequently used for γδ T cell culture. Paraffin‑embedded tissue samples were obtained from 10 patients with esophageal carcinoma (ESCA) who underwent surgical resection at the First Affiliated Hospital of Henan Medical University. None of the patients had received any systemic or local therapy prior to surgery. The study protocol was approved by the Ethics Committee of Xinxiang Medical University (Approval No. XYLL-20240393). Written informed consent was obtained from all participants (both healthy volunteers and patients) before sample collection.

### Esophageal cancer cell culture

Esophageal cancer cell lines TE-1 and TE-10 were preserved in the laboratory. Cells were cultured in RPMI 1640 medium (Gibco) supplemented with 10% fetal bovine serum (AusgeneX), 1% penicillin-streptomycin double antibody under standard conditions (37°C, 5% CO2). Subculture was performed when cell confluence reached 80%. All experiments were performed during the exponential phase of cell growth.

### Human Vγ9Vδ2 T cell culture

The in vitro expansion of γδ T cells was performed according to our established protocol [15]. Briefly, whole blood was diluted twofold with serum-free RPMI 1640 medium and carefully layered over an equal volume of human lymphocyte separation medium in a 50 mL conical tube. The samples were centrifuged at 600 × g for 25 min at RT. Following centrifugation, the buffy coat layer containing PBMCs was aspirated using a sterile Pasteur pipette and transferred to a new tube. The cells were washed with serum-free RPMI 1640 medium and centrifuged at 500 × g for 15 min. Erythrocyte contamination was removed by resuspending the cell pellet in 2-3 mL of red blood cell lysis buffer for 5 min at room temperature, followed by the addition of three volumes of serum-free RPMI 1640 medium to neutralize the reaction. The cell suspension was filtered through a 40 μm cell strainer and centrifuged at 500 × g for 10 min. After an additional wash step, the purified PBMCs were resuspended in complete RPMI 1640 medium supplemented with 10% fetal bovine serum and counted using a hemocytometer. For the expansion of Vγ9Vδ2 T cells, PBMCs were seeded at a density of 3.5 × 10^6^ cells/mL in complete medium containing 10% FBS and stimulated with zoledronic acid (Sigma-Aldrich) and 100 IU/mL recombinant human IL-2 (Sihuan Biotechnology) on day 0. On day 3, the cell density was adjusted to 3 × 10^6^ cells/mL with fresh complete medium supplemented with IL-2 alone. Thereafter, the cell density was maintained at 3 × 10^6^ cells/mL until day 6, 2 × 10^6^ cells/mL on day 7, and 1 × 10^6^ cells/mL from day 9 onwards, with medium replenishment every 48 h. The purity of Vγ9Vδ2 T cells was assessed by flow cytometry between days 9–11, and cultures exhibiting >85% Vδ2 positivity were used for subsequent functional experiments.

### Western blot

Total proteins were extracted from metformin- or Compound C-treated TE-1 and TE-10 cells with RIPA lysis buffer (Beyotime) containing protease and phosphatase inhibitors (Yamei Biotechnology). The protein samples were separated by SDS-polyacrylamide gel electrophoresis and subsequently transferred onto PVDF membranes. Following transfer, the membranes were blocked with 5% non-fat milk for 1 h and then incubated overnight at 4 °C with the following primary antibodies: anti-BTN2A1 (1:1000, Proteintech), anti-BTN3A1 (1:1000, Proteintech), and anti-GAPDH (1:5000, Proteintech). After thorough washing, the membranes were probed with appropriate horseradish peroxidase (HRP)-conjugated secondary antibodies (1:3500, CWBIO) for 1 h at room temperature. Protein bands were visualized using an enhanced chemiluminescence substrate prepared by mixing Solution A and Solution B at a 1:1 ratio, and images were captured with a Tanon-5200Multi imaging system. The band intensity was quantified by grayscale analysis using ImageJ software.

### Flow cytometry analysis

TE-10 and TE-1 esophageal cancer cells were seeded in 6 cm dishes and treated with metformin (MCE) at varying concentrations (TE-10: 0, 0.0625, 0.125 mmol/L; TE-1: 0, 0.125, 0.25 mmol/L) for 24 h upon reaching 60% confluence. Harvested cells were stained with 2 μL BTN3A1 antibody (BioLegend) and 2 μL BTN2A1 antibody (CUSABIO) at 4 °C for 15 min, the proportions of BTN3A1-positive and BTN2A1-positive cells were quantified by flow cytometry. To verify the direct effect of metformin on Vγ9Vδ2 T cell proliferation and function, we performed functional immune assays. Vγ9Vδ2 T cells were pretreated with metformin for 4 hours. Following metformin pretreatment, we assessed T cell proliferation by Ki67 staining, cytokine production (IFN‑γ and TNF‑α), activation marker expression (CD69), and cytotoxic potential (CD107a) using flow cytometry. For the co-culture assay, Vγ9Vδ2 T cells (effectors) were co-cultured with metformin-treated or untreated tumor cells at an E:T ratio of 10:1 for 3.5 h. Subsequently, cells were centrifuged, resuspended in 100 μL PBS, and stained with 1 μL Vδ2 antibody plus 2 μL of CD69 or CD107a antibodies (BioLegend) at 4 °C for 20 min. The frequencies of CD69- or CD107a-positive Vγ9Vδ2 T cells were determined via flow cytometry.

### Effector-to-Target ratio analysis

TE-1 and TE-10 esophageal cancer cells were harvested, washed twice with PBS, and resuspended in 4 mL PBS containing 0.2 μL CFSE (Sigma) for 50 s at 37 °C in the dark. The staining reaction was quenched by adding 4 mL of complete medium. Labeled tumor cells were co-cultured with Vγ9Vδ2 T cells in flow tubes at effector-to-target (E:T) ratios of 0:1, 1:1, 10:1, and 20:1 for 6 h at 37 °C. After co-culture, cells were centrifuged and stained with 2 μL propidium iodide (PI; BioLegend) in 100 μL PBS at 4 °C for 10 min. Vγ9Vδ2 T cell-mediated killing efficiency was assessed by flow cytometry based on CFSE and PI staining.

### In vitro cytotoxicity assay

TE-1 and TE-10 cells, either untreated or pre-treated with metformin, were harvested and labeled with CFSE. Briefly, cell pellets were resuspended in 4 mL PBS containing 0.2 μL CFSE and incubated at 37 °C in the dark for 50 s. The staining was quenched by adding 4 mL of complete medium. Then, 2 × 10^5^ labeled tumor cells were aliquoted into flow cytometry tubes and assigned to the following experimental groups: (1) tumor cells alone (Control), (2) tumor cells with metformin only, (3) tumor cells with Vγ9Vδ2 T cells only, and (4) tumor cells with both metformin and Vγ9Vδ2 T cells (Combination). Metformin was applied at 0.25 mmol/L for TE-1 and 0.125 mmol/L for TE-10 cells. Vγ9Vδ2 T cells were co-cultured at an effector-to-target (E:T) ratio of 10:1. After 6 h of incubation at 37 °C, cells were pelleted and stained with 2 μL propidium iodide (PI) in 100 μL PBS at 4 °C for 10 min. Vγ9Vδ2 T cell-mediated killing efficiency was determined by flow cytometry based on CFSE and PI double-staining.

### Cytotoxicity assay with BTN3A1 blockade

Tumor cells grown under normal conditions or treated with metformin were collected, centrifuged, washed twice with PBS, and the supernatant was discarded. The cell pellet was resuspended in 4 mL of PBS containing 0.2 μL of CFSE and incubated at 37 °C in the dark for 50 s. Staining was terminated by adding 4 mL of complete medium. Then, 2 × 10^5^ tumor cells were aliquoted into different flow tubes and divided into five groups: control group (no treatment), Vγ9Vδ2 T cell treatment group, Vγ9Vδ2 T cell treatment group with BTN3A1 antibody blockade, combination treatment group, and combination treatment group with BTN3A1 antibody blockade. The E:T ratio was 10:1 for Vγ9Vδ2 T cells and target cells. The antibody blockade groups were added with 4 μL of BTN3A1 blocking antibody (BioLegend). After co-incubation in a 37 °C constant temperature incubator for 6 hours, cells were centrifuged, and the pellet was resuspended in 100 μL of PBS containing 2 μL of PI, followed by staining at 4 °C for 10 min. After terminating the staining, the killing efficiency of Vγ9Vδ2 T cells was detected by flow cytometry.

### ELISA assay

Each well of the plate was coated with 100 μL of diluted capture antibody and incubated overnight at 4 °C. After four washes, 100 μL of diluted standards or test samples (supernatants from Vγ9Vδ2 T cell–tumor cell co-cultures) were added to designated wells. The plate was sealed and incubated with shaking at room temperature for 2 h. Following another four washes, 100 μL of diluted detection antibody was added per well, and the plate was shaken for 1 h at room temperature. After repeating the washing step, 100 μL of diluted Avidin-HRP was added and incubated for 30 min at room temperature. The plate was then washed five times, followed by addition of 100 μL TMB substrate and incubation in the dark for 15 minutes. The reaction was stopped with 100 μL stop solution per well. Absorbance was immediately measured at 450 nm with 570 nm as the reference wavelength. Expression levels of relevant effector molecules were determined using the standard curve.

### Animal model and therapeutic efficacy assessment

Female NOD/SCID mice (6–8 weeks old, 19–23 g) were obtained from Suzhou Cyagen Biosciences and maintained under specific pathogen-free conditions (temperature: 22 ± 2 °C, humidity: 40–50%, 12-hour light/dark cycle) with free access to food and water. Mice were randomly assigned into four experimental groups (*n* = 4 per group): (1) Control (PBS), (2) Metformin alone, (3) Vγ9Vδ2 T cells alone, and (4) Metformin + Vγ9Vδ2 T cells (Combination).

Esophageal cancer cells (5 × 10^6^ TE-1 or 1 × 10^7^ TE-10 cells per mouse) were inoculated subcutaneously into the lower dorsal flank. Treatment commenced upon palpable tumor formation. Mice in the metformin and combination groups received intraperitoneal (i.p.) injections of metformin (200 mg/kg) as previously described daily for 15 consecutive days [23, 32]. Mice in the Vγ9Vδ2 T cell and combination groups received i.v. injections of Vγ9Vδ2 T cells (5 × 10^6^ cells per injection) every 3 days for a total of 5 injections. Control mice received an equal volume of PBS on a matching schedule.

Body weight and tumor dimensions were monitored regularly. Tumor volume was calculated using the formula: Volume = (a × b^2^)/2, where a is the longest tumor diameter and b is the shortest diameter. At the experimental endpoint, mice were euthanized, and tumor, kidney, and liver tissues were harvested for subsequent analysis.

### Hematoxylin-Eosin (HE) staining

Tissue sections were routinely stained with hematoxylin and eosin (H&E). Briefly, sections were stained with hematoxylin for 5 min, followed by eosin for 1 min. Subsequently, the slides were dehydrated through a graded ethanol series, cleared in xylene, and mounted with neutral balsam. Stained tissues were examined and imaged under a microscope.

### Immunohistochemistry

Tumor tissues were fixed in 4% paraformaldehyde, embedded in paraffin, and sectioned. Following deparaffinization and antigen retrieval, endogenous peroxidases were blocked by applying Hydrogen Peroxide Blocking Reagent (Vazyme H&R HRP/DAB Detection IHC Kit) for 20 min at room temperature. Sections were then incubated overnight at 4 °C with primary antibodies against BTN3A1 (1:100, Bioworld Biotech) or BTN2A1 (1:100, Bioss). After rewarming and washing, HRP Polymer was applied for 20 min at room temperature. Antigen-antibody binding was visualized using 3,3’-diaminobenzidine (DAB) substrate, with the reaction monitored microscopically and stopped upon the development of brown signal. Finally, sections were counterstained with hematoxylin, dehydrated through a graded ethanol series, cleared in xylene, and mounted with neutral balsam. Stained images were acquired using a light microscope.

### Immunofluorescence

Tissue sections were probed overnight at 4 °C in the dark with a cocktail of primary antibodies, including anti-Vδ2 (1:150, BioLegend) and anti-Granzyme B (1:150, Proteintech), prepared according to the manufacturers’ instructions. The following day, sections were washed and incubated with a species-appropriate fluorescent secondary antibody (Proteintech, Cat# SA00003-2) for 1 h at room temperature in the dark. Following PBS washes, nuclei were counterstained with DAPI for 8 min. Finally, sections were mounted with an anti-fade mounting medium and imaged using a confocal laser scanning microscope.

### One-Step TUNEL assay

Apoptosis in tissue sections was detected using the TUNEL assay kit (Beyotime) according to the manufacturer’s protocol. Briefly, the TUNEL reaction mixture was prepared by mixing TdT enzyme and fluorescent labeling solution at a 1:9 volume ratio. The mixture was applied to cover each section and incubated at 37 °C in the dark for 60 min. Following incubation, nuclei were counterstained with DAPI (Service bio) for 8 min at room temperature. After a final wash with PBS, sections were mounted with an anti-fade mounting medium (Service bio) and imaged under a laser scanning confocal microscope.

### Statistical analysis

GraphPad Prism 8.0 was used for all analyses. Data are shown as mean ± SD. Group comparisons were performed using an unpaired Student’s t-test (two groups) or one-way ANOVA with Tukey’s post hoc test (multiple groups). *p < 0.05 was considered significant.

## Results

### Metformin upregulates the expression of BTN3A1 and BTN2A1 in esophageal cancer cells

**Fig. 1:**
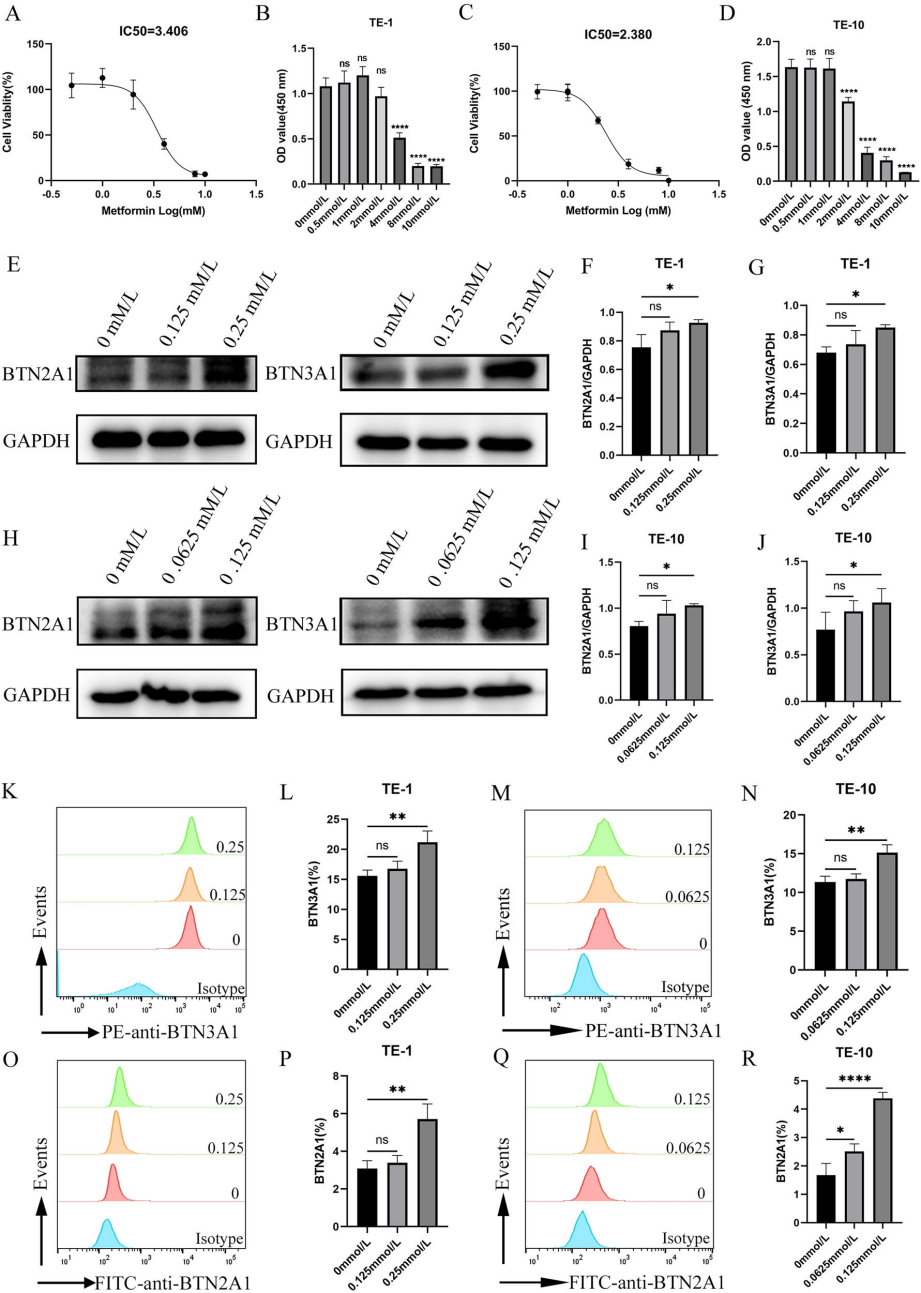
Metformin upregulates the expression of BTN3A1 and BTN2A1 in human esophageal cancer cells. (**A**-**D**) Dose-response curves of esophageal cancer cell lines TE-1 and TE-10 treated with the indicated concentrations of metformin for 48 hours. Cell viability was assessed to determine the half-maximal inhibitory concentration (IC_50_). Data are presented as mean ± SD (*n* = 5 independent experiments). (**E**-**J**) Western blot analysis of BTN2A1 and BTN3A1 protein expression in TE-1 and TE-10 cells treated with or without metformin for 24 hours. GAPDH served as the loading control. Representative blots are shown (**E**, **H**). Densitometric quantification of BTN2A1 (**F**, **G**) and BTN3A1 (**I**, **J**) protein levels normalized to GAPDH is presented as mean ± SD (*n* = 3). *p < 0.05, **p < 0.01, ***p < 0.001 versus the respective control group (0 mM metformin); one-way ANOVA with Dunnett’s post-hoc test. (**K**-**R**) Flow cytometric analysis of cell surface BTN3A1 (panels K-N) and BTN2A1 (panels O-R) expression in TE-1 and TE-10 cells treated with or without metformin for 24 hours. For BTN3A1: representative histogram overlays are shown TE-1 (**K**) and TE-10 (**M**); the geometric mean fluorescence intensity (gMFI) was quantified and is presented as mean ± SD (*n* = 3 independent experiments) for TE-1 (L) and TE-10 (N). For BTN2A1: representative histograms are shown for TE-1 (**O**) and TE-10 (**Q**); gMFI quantification is shown for TE-1 (**P**) and TE-10 (**R**). *p < 0.05, **p < 0.01, ***p < 0.001 versus the respective control group (0 mM metformin); one-way ANOVA with Dunnett’s post-hoc test.

Metformin exerts antitumor effects through established mechanisms, including AMPK activation and mTOR inhibition, alongside broader immunomodulatory properties [33]. However, its direct impact on the expression of BTN3A1 and BTN2A1—key ligands for γδ T cell activation—remained unclear. To investigate this, we first determined the half-maximal inhibitory concentration (IC_50_) of metformin in esophageal cancer cell lines TE-1 and TE-10 after 48 hours of treatment. The resulting IC_50_ values were determined as 3.406 mM for TE-1 and 2.38 mM for TE-10 (Fig. 1A–D). Based on these values, cells were treated with a concentration gradient of metformin for 24 h. In TE-1 cells, BTN2A1 and BTN3A1 protein expression increased in a dose-dependent manner at concentrations up to 0.25 mM. Notably, TE-10 cells also exhibited this dose-dependent upregulation, with a marked induction at 0.125 mM (Fig. 1E-J and Supplementary Fig. 1A, D and E). Furthermore, flow cytometry confirmed a significant increase in the surface presentation of both BTN3A1 and BTN2A1 following metformin treatment, corroborating the western blot findings (Fig. 1K-R). To determine whether this regulation extended to other butyrophilin family members implicated in γδ T cell activation, we assessed BTN3A2 and BTN3A3 expression by qPCR. In TE-1 cells, metformin did not significantly alter the expression of either BTN3A2 or BTN3A3. In TE-10 cells, BTN3A2 expression remained unchanged, whereas BTN3A3 was significantly upregulated, indicating isoform- and cell line‑specific effects (Supplementary Fig. 2). To further demonstrate the mechanism of metformin, we treated cells with a well‑established AMPK inhibitor (Compound C) and found that it significantly suppresses metformin‑induced upregulation of both BTN3A1 and BTN2A1, indicating that metformin acts, at least in part, through the AMPK pathway to regulate these genes (Supplementary Fig. 3). Collectively, these results demonstrate that metformin upregulates BTN3A1 and BTN2A1 in esophageal cancer cells through an AMPK-dependent pathway, revealing a novel immunomodulatory mechanism by which metformin may enhance γδ T cell-mediated antitumor immunity.

### Metformin synergizes with Vγ9Vδ2 T cells to enhance cytotoxicity against esophageal cancer cells in vitro

**Fig. 2:**
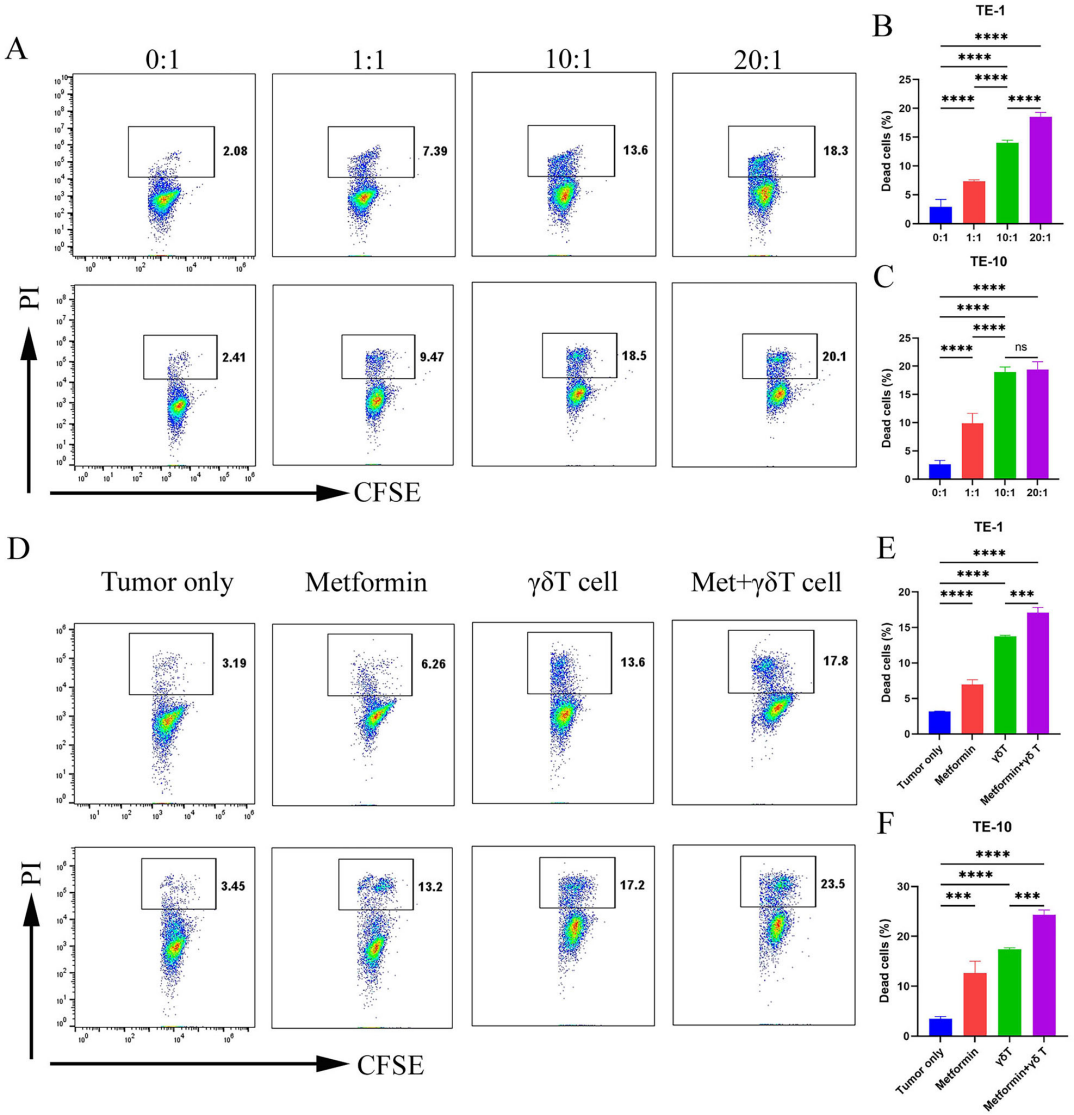
Metformin synergizes with Vγ9Vδ2 T cells to enhance cytotoxicity against esophageal cancer cells in vitro. (**A**-**C**) Cytotoxic activity of Vγ9Vδ2 T cells against esophageal cancer cell lines TE-1 (**A**, **B**) and TE-10 (**A**, **C**) following pretreatment with or without metformin. Target cells were co-cultured with Vγ9Vδ2 T cells at the indicated effector-to-target (E:T) ratios (0:1, 1:1, 10:1, and 20:1) for 6 hours. Specific tumor cell lysis was determined using flow cytometry based on CFSE and PI staining. Data are presented as mean ± SD (*n* = 3 independent experiments). Statistical analysis was performed using one-way ANOVA with Tukey’s post-hoc test. (**D**-**F**) Comparison of the antitumor efficacy of metformin monotherapy, Vγ9Vδ2 T cell monotherapy, and their combination. TE-1 (**D**, **E**) and TE-10 (**D**, **F**) cells were treated with metformin, Vγ9Vδ2 T cells (E:T = 10:1), or the combination of both for 6 hours. Specific lysis was quantified. Data are presented as mean ± SD (*n* = 3 independent experiments). Statistical analysis was performed using one-way ANOVA with Tukey’s post-hoc test. **p < 0.01, ***p < 0.001, ****p < 0.0001 for the indicated comparisons.

Building upon our previous finding that metformin upregulates the γδ T cell-stimulating ligands BTN3A1 and BTN2A1, we sought to determine whether this molecular alteration functionally enhances Vγ9Vδ2 T cell-mediated cytotoxicity against esophageal cancer cells. To establish a robust experimental system, we first titrated the effector-to-target (E:T) ratio using TE-1 and TE-10 cell lines. Through quantitative analysis of tumor cell lysis, we found that E:T ratios of 10:1 and 20:1 induced significantly superior cytotoxicity compared to both untreated controls and the 1:1 ratio (*p* < 0.001), with specific killing rates reaching 14.03% ± 0.43% and 18.5% ± 0.8% in TE-1 cells, and 18.98% ± 0.88% and 19.4% ± 1.43% in TE-10 cells, respectively (Fig. 2A-C). Based on this dose-response relationship and considerations of clinical applicability, we selected the 10:1 E:T ratio for all subsequent combination experiments.

We next performed comprehensive cytotoxicity assessments across four experimental conditions: untreated control, metformin monotherapy (24-hour pretreatment), Vγ9Vδ2 T cells alone, and the combination of metformin pretreatment followed by Vγ9Vδ2 T cell co-culture. Strikingly, the combination treatment resulted in dramatically enhanced tumor cell mortality (17.07% ± 0.75% in TE-1; 24.3% ± 0.98% in TE-10) compared to either treatment alone (metformin monotherapy: 6.96% ± 0.68% in TE-1, 12.67% ± 2.35% in TE-10; Vγ9Vδ2 T cells alone: 13.73% ± 0.15% in TE-1, 17.43% ± 0.25% in TE-10; *p* < 0.0001 for all comparisons) (Fig. 2D-F). These results demonstrate that metformin functionally synergizes with Vγ9Vδ2 T cells and provide compelling experimental evidence that its previously observed upregulation of BTN3A1/ BTN2A1 contributes to enhanced γδ T cell-mediated antitumor immunity.

### Blockade of BTN3A1 reverses metformin-enhanced Vγ9Vδ2 T cell cytotoxicity

**Fig. 3:**
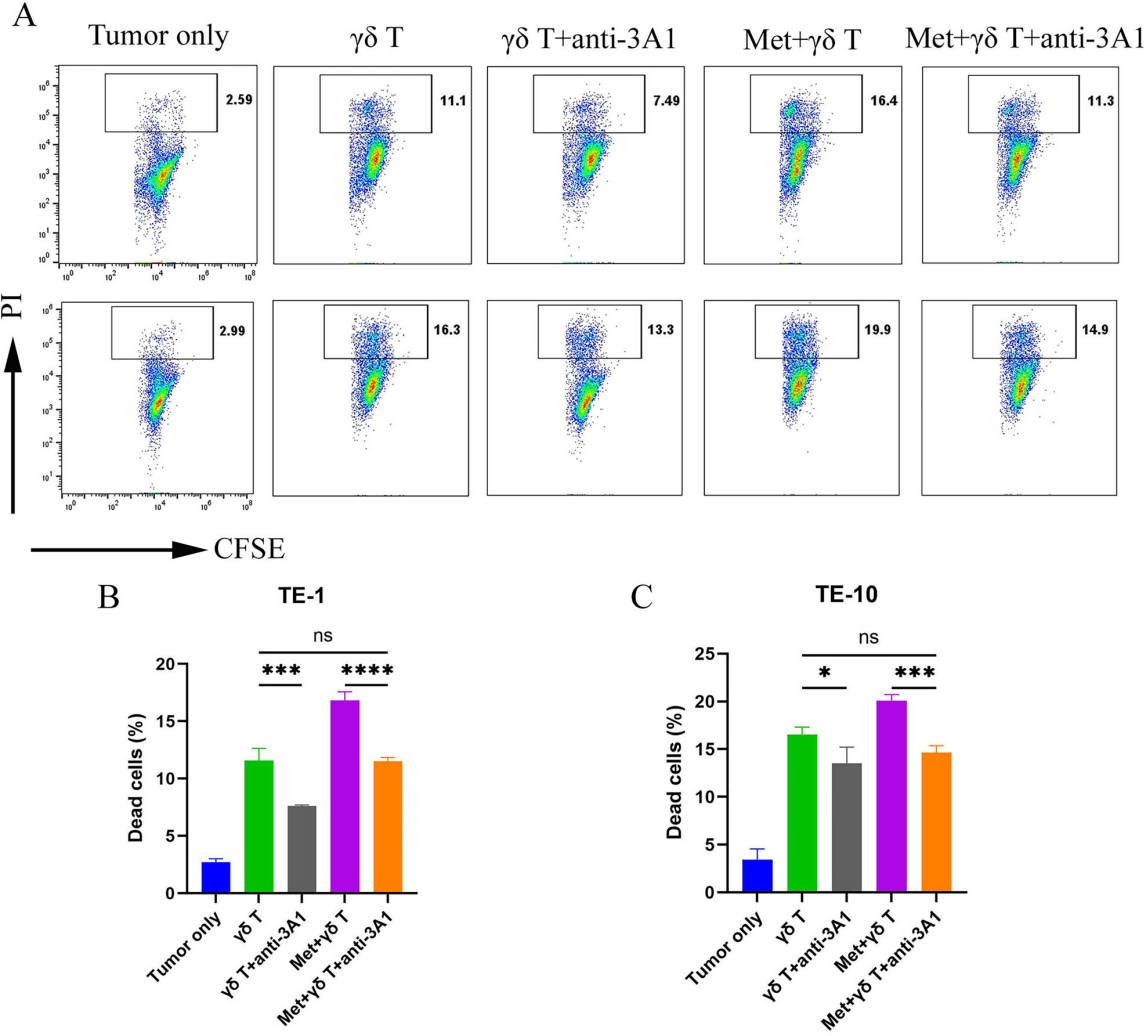
Blockade of BTN3A1 reverses metformin-enhanced Vγ9Vδ2 T cell cytotoxicity. (**A**) Flow cytometric analysis of tumor cell death (TE-1 and TE-10 lines) following co-culture with Vγ9Vδ2 T cells under indicated conditions. Cells were treated with or without metformin, and BTN3A1-blocking antibody was added during the co-culture. Tumor cell cytotoxicity was assessed by measuring specific killing. (B, C) Quantification of Vγ9Vδ2 T cell-mediated cytotoxicity against TE-1 (**B**) and TE-10 (**C**) cells, corresponding to the flow cytometry data in (A). Data are presented as mean ± SEM of specific lysis. Statistical significance was determined by one-way ANOVA with appropriate post-hoc tests (*p < 0.05, **p < 0.01, ***p < 0.001; ns, not significant). Results are representative of at least three independent experiments.

Having established that metformin upregulates BTN3A1/BTN2A1 and synergizes with Vγ9Vδ2 T cells, we next sought to determine if these phenomena are mechanistically linked. We hypothesized that if the synergy is mediated via this complex, then blocking BTN3A1 should reverse the enhanced killing. Indeed, introducing a function-blocking anti-BTN3A1 antibody during co-culture significantly reduced the cytotoxicity of Vγ9Vδ2 T cells alone (Fig. 3). More importantly, it effectively abolished the additional killing potency conferred by metformin pre-treatment, returning cytotoxicity to the level observed with Vγ9Vδ2 T cells alone (Fig. 3). This result positions the BTN2A1-BTN3A1 complex as the essential functional bridge between metformin’s molecular action and its potentiation of γδ T cell immunity, highlighting a targetable axis for therapeutic intervention.

### Metformin licenses a superior effector phenotype in Vγ9Vδ2 T cells

**Fig. 4:**
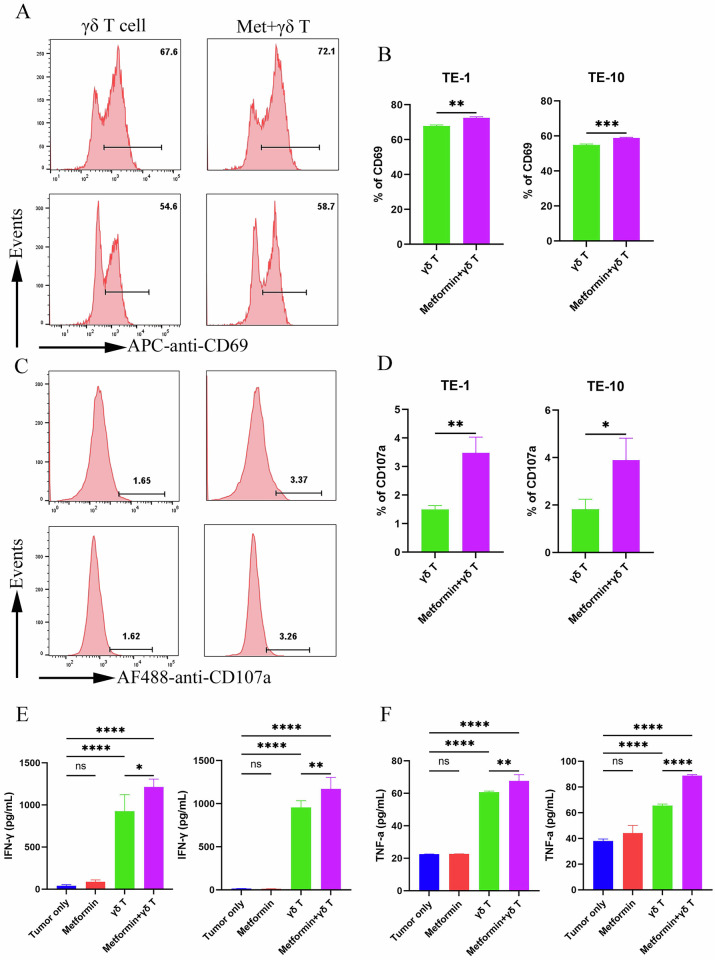
Metformin licenses a superior effector phenotype in Vγ9Vδ2 T cells. (**A**, **C**) Representative flow cytometry plots depicting the expression of the early activation marker CD69 (A) and the degranulation marker CD107a (C) on Vδ2^+^ T cells after a 3.5-hour co-culture with either untreated or metformin-pretreated tumor cells at an effector-to-target (E:T) ratio of 10:1. All plots shown in (**A**, **C**) are gated on Vδ2^+^ lymphocytes. (**B**, **D**) Quantification of CD69 (**B**) and CD107a (**D**) expression levels gated on Vδ2^+^ T cells, presented as mean fluorescence intensity (MFI) or percentage of positive cells. Data correspond to the representative experiments shown in (**A**) and (C). (**E**, **F**) Secreted levels of interferon-γ (IFN-γ, E) and tumor necrosis factor-α (TNF-α, F) in the co-culture supernatants, as measured by enzyme-linked immunosorbent assay (ELISA). Cytokine production by Vγ9Vδ2 T cells was assessed following co-culture with the indicated tumor cell conditions. Data in bar graphs (**B**, **D**, **E**, **F**) are presented as mean ± SEM from at least three independent experiments. Statistical significance was determined by Student’s t-test or one-way ANOVA (*p < 0.05, **p < 0.01, ***p < 0.001; ns, not significant).

Building on our findings that metformin upregulates the BTN2A1-BTN3A1 complex and synergistically enhances tumor cell killing, we next asked whether this was accompanied by improved Vγ9Vδ2 T cell functionality. We hypothesized that increased ligand engagement would drive T cell activation. Consistent with this, the combination treatment significantly increased the expression of the early activation marker CD69 on Vγ9Vδ2 T cells compared to their state in monotherapy. (Fig. 4A, B) To comprehensively assess the functional corollary, we also measured degranulation (via CD107a membrane exposure) and cytokine production (IFN-γ and TNF-α). The results demonstrated a coordinated enhancement: metformin-treated co-cultures showed significantly elevated frequencies of CD107a^+^ T cells (Fig. 4C, D) and increased production of both cytokines (Fig. 4E, F). Importantly, we also assessed whether metformin directly modulates γδ T cell function in the absence of tumor cells. Following metformin pretreatment, flow cytometry analysis revealed no significant changes in proliferation (Ki67), activation (CD69), degranulation (CD107a), or cytokine production (IFN-γ and TNF-α), indicating that metformin does not exert direct effects on γδ T cells under these conditions (Supplementary Fig. 3). This multi-parameter immune profiling confirms that the synergy originates from metformin-induced immunomodulation, which augments both the recognition and effector phases of Vγ9Vδ2 T cell-mediated antitumor immunity.

### Metformin Synergizes with Vγ9Vδ2 T Cells to Suppress Tumor Growth In Vivo

**Fig. 5:**
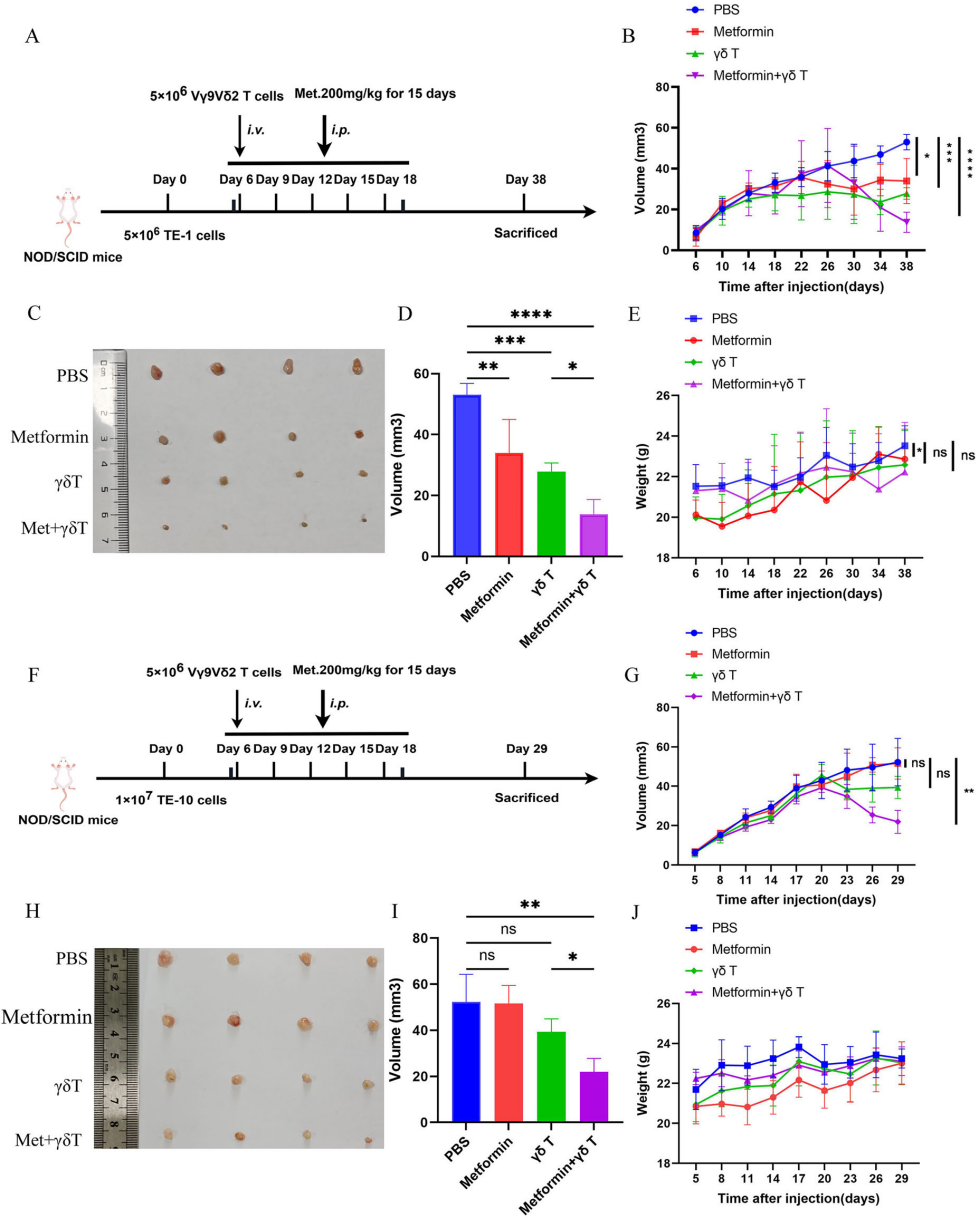
Metformin Synergizes with Vγ9Vδ2 T Cells to Suppress Tumor Growth In Vivo. (**A**) Schematic of the therapeutic regimen in the TE-1 ectopic xenograft model. Mice were inoculated with TE-1 tumor cells, followed by administration of Metformin, Vγ9Vδ2 T cells, or their combination as indicated. (**B**) Tumor growth curves of TE-1 xenografts over time. Tumor volume was measured at the indicated time points following the initiation of treatments. Data are presented as mean ± SEM. (**C**) Photographs of excised TE-1 tumors from each treatment group at the experimental endpoint. (**D**) Quantification of tumor volume from the TE-1 model at endpoint. Bars represent mean ± SEM. (**E**) Body weight changes of mice bearing TE-1 tumors during the treatment period, monitored as an indicator of systemic toxicity. (**F**) Schematic of the therapeutic regimen in the TE-10 ectopic xenograft model. (**G**) Tumor growth curves of TE-10 xenografts over time, presented as mean tumor volume ± SEM. (**H**) Photographs of excised TE-10 tumors from each treatment group at endpoint. (**I**) Quantification of tumor volume from the TE-10 model at endpoint. Bars represent mean ± SEM. (**J**) Body weight changes of mice bearing TE-10 tumors during the treatment period. For panels B and G, statistical significance of tumor growth curves was analyzed by two-way ANOVA with repeated measures. For panels D and I, statistical significance was determined by one-way ANOVA followed by appropriate post-hoc tests (*p < 0.05, **p < 0.01, ***p < 0.001, *n* = 4 mice per group per experiment).

Based on our compelling in vitro evidence that metformin enhances Vγ9Vδ2 T cell cytotoxicity through upregulation of the BTN3A1/BTN2A1 axis, we next evaluated the translational potential of this combination in vivo. Xenograft models were established in NOD/SCID mice by subcutaneous inoculation with TE-1 or TE-10 esophageal cancer cells. Mice were randomized into four treatment groups: vehicle control, metformin monotherapy (200 mg/kg, i.p., daily), Vγ9Vδ2 T cell monotherapy (5×10^6^ cells, i.v., administered on days 6, 9, 12, 15 and 18), and the combination of metformin and Vγ9Vδ2 T cells (Figs. 5A and F). Tumor growth was monitored until the experimental endpoint (Figs. 5D and I).

The combination treatment resulted in profound and synergistic suppression of tumor growth in both models. In TE-1-bearing mice, tumor growth inhibition became statistically significant from day 34 onward, with the combination group exhibiting the most pronounced reduction in final tumor volume (Fig. 5B, C). In the more aggressive TE-10 model, the combination therapy significantly suppressed tumor progression as early as day 23, and continued to potently inhibit tumor growth until the animals were sacrificed. (Fig. 5G, H). Notably, metformin monotherapy alone demonstrated a modest but consistent antitumor effect, confirming its intrinsic activity in TE-1 model (Fig. 5D). Importantly, this potent synergistic effect was achieved without observable systemic toxicity, as evidenced by stable body weights across all treatment groups and normal morphology in liver and kidney tissues (Supplementary Fig. 2). These robust in vivo findings conclusively demonstrate that metformin-induced BTN3A1/BTN2A1 upregulation functionally sensitizes esophageal tumors to Vγ9Vδ2 T cell-mediated clearance, positioning this combination as a potent and well-tolerated immunotherapeutic strategy.

### Metformin upregulates BTN3A1/BTN2A1 to promote Vγ9Vδ2 T cell tumor cytotoxic function

**Fig. 6:**
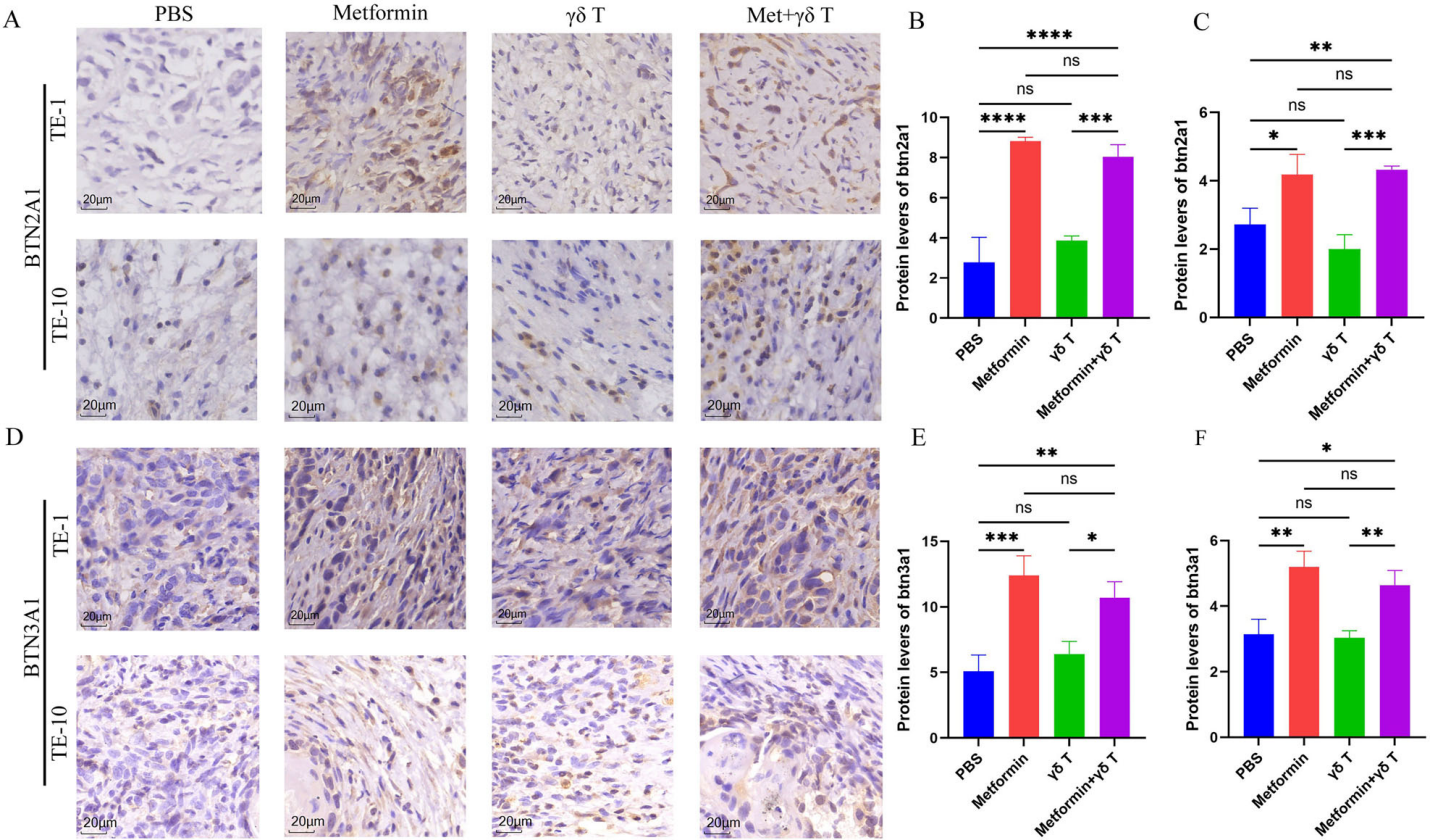
Metformin upregulates the expression of BTN2A1 and BTN3A1 in tumor tissues in vivo. (**A**) Representative immunohistochemistry (IHC) images showing BTN2A1 protein expression in excised TE-1 and TE-10 tumor tissues from the indicated treatment groups (Scale bar, 20 μm). (**B**, **C**) Quantitative analysis of BTN2A1 IHC staining intensity in TE-1 (**B**) and TE-10 (**C**) tumor tissues. Expression levels are presented as mean staining scores ± SEM. (**D**) Representative IHC images showing BTN3A1 protein expression in excised TE-1 and TE-10 tumor tissues from the indicated treatment groups (Scale bar, 20 μm). (**E**, **F**) Quantitative analysis of BTN3A1 IHC staining intensity in TE-1 (**E**) and TE-10 (**F**) tumor tissues. Expression levels are presented as mean staining scores ± SEM. IHC staining was evaluated and scored in a blinded manner. For quantification, multiple fields from at least 3 tumors per group were analyzed. Statistical significance was determined by one-way ANOVA followed by appropriate post-hoc tests (*p < 0.05, **p < 0.01, **p < 0.001).

Building upon our in vivo demonstration of tumor growth suppression, we sought to elucidate the underlying mechanisms by examining molecular and cellular changes within the tumor microenvironment. Analysis of tumor sections from xenograft models revealed a consistent pattern: while Vγ9Vδ2 T cell monotherapy alone did not significantly alter BTN3A1/BTN2A1 expression compared to PBS group, both metformin monotherapy and the combination treatment markedly upregulated these γδ T cell-stimulating ligands (Fig. 6). The enhancement in the combination group was quantitatively comparable to metformin alone, confirming the drug’s primary role in modulating this molecular axis.

**Fig. 7:**
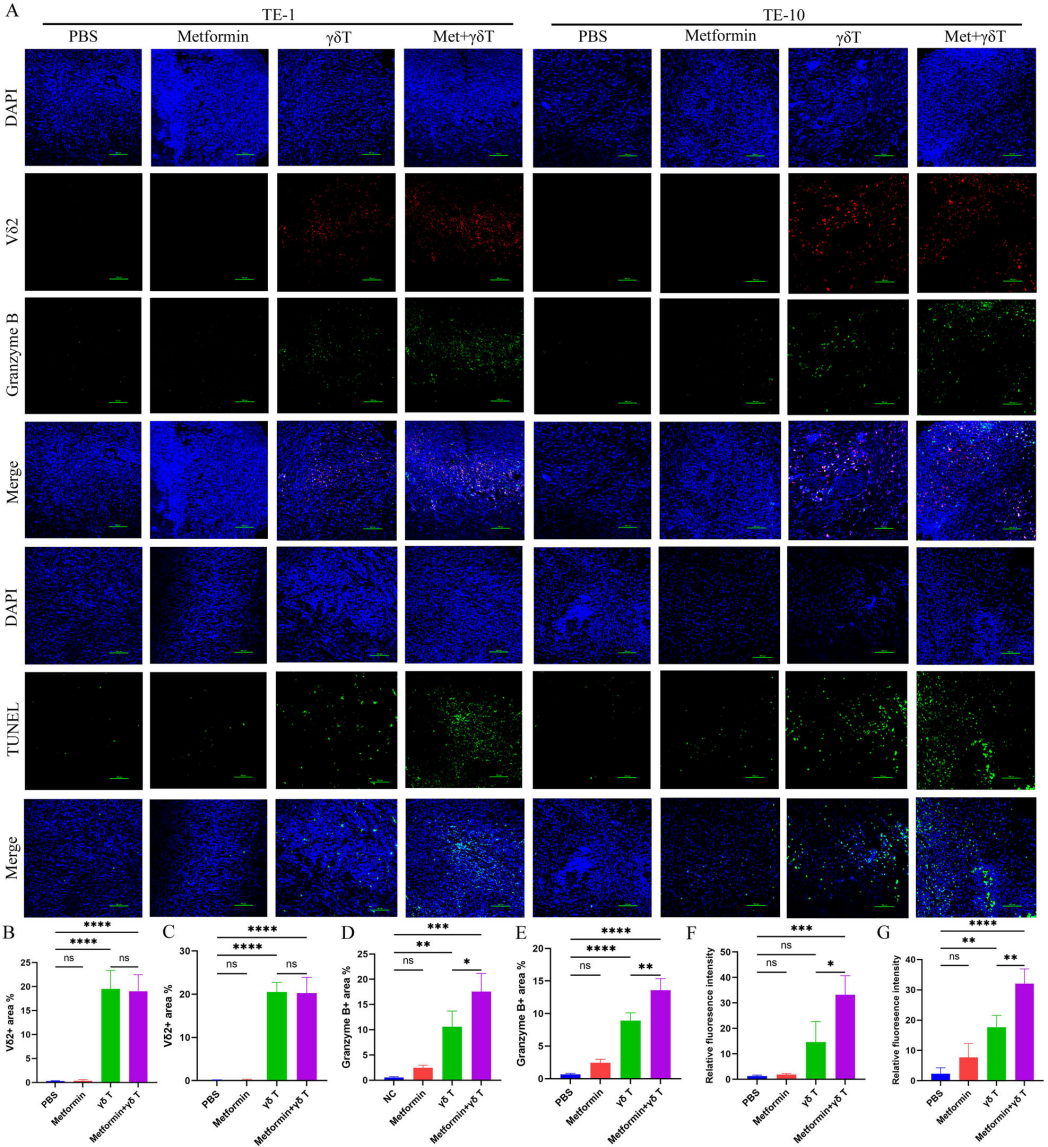
Metformin combined with Vγ9Vδ2 T cells enhances intratumoral T cell activation, and tumor cell apoptosis in vivo. **A** Representative immunofluorescence (IF) images of tumor tissues (TE-1 and TE-10) from different treatment groups. Vγ9Vδ2 T cell infiltration was detected using an anti-Vδ2 antibody (red), and their activation status was assessed by co-staining for Granzyme B (GrB, green). Cell nuclei were counterstained with DAPI (blue). Scale bar, 100 μm. **B**, **C** Quantification of Vδ2^+^ T cell infiltration within TE-1 **B** and TE-10 **C** tumors. Data are presented as the number of Vδ2^+^ cells per high-power field (HPF). **D**, **E** Quantification of activated, GrB-producing cells within TE-1 **D** and TE-10 **E** tumors. Data are presented as the number of GrB^+^ cells per HPF. **F**, **G** Quantification of tumor cell apoptosis assessed by TUNEL assay in TE-1 **F** and TE-10 **G** tumor sections. Data are presented as the percentage of TUNEL- positive (apoptotic) cells per field. For all quantifications **B**–**G**, multiple random fields from at least 3 tumors per group were analyzed in a blinded manner. Data are presented as mean ± SEM. Statistical significance was determined by one-way ANOVA followed by appropriate post-hoc tests (*p < 0.05, **p < 0.01, ***p < 0.001).

We next investigated whether this metformin-induced ligand upregulation translated to improved immune cell recruitment. Quantification of TCRγδ+ cells by immunofluorescence staining revealed that Vγ9Vδ2 T cell infiltration was significantly increased in both the Vγ9Vδ2 T cell monotherapy group and the combination treatment group compared to control or metformin-alone groups (Fig. 7A–C). Notably, there was no statistically significant difference in the extent of infiltration between the Vγ9Vδ2 T cell monotherapy and combination groups. These results indicate that metformin, while upregulating BTN3A1/BTN2A1, does not enhance the tumor-homing capacity of Vγ9Vδ2 T cells on its own, and that the adoptively transferred T cells are capable of infiltrating tumors independently of metformin pretreatment.

To assess the functional consequences of this immunomodulation, we evaluated cytotoxic activity within tumor tissues. Analysis of Granzyme B (GZMB) revealed that significant increases in intratumoral GZMB deposition were specifically observed in the Vγ9Vδ2 T cell monotherapy and combination groups, with the combination treatment inducing the most substantial enhancement (Fig. 7A, D, E). Furthermore, TUNEL assays demonstrated a markedly elevated apoptotic response in the combination group. Although Vγ9Vδ2 T cell monotherapy alone significantly increased tumor cell apoptosis compared to control groups, the combination with metformin resulted in a substantially greater effect, yielding approximately 2.32 and 1.82-fold more TUNEL-positive cells than Vγ9Vδ2 T cells alone (Fig. 7A, F, G). This widespread apoptosis was predominantly localized to tumor regions with dense Vγ9Vδ2 T cell infiltration.

These results provide a mechanistic continuum from metformin-induced BTN3A1/BTN2A1 upregulation through enhanced Vγ9Vδ2 T cell trafficking to potentiated cytotoxic function, ultimately explaining the superior antitumor efficacy observed in our in vivo models.

### BTN3A1 and BTN2A1 upregulation in esophageal cancer: a potential biomarker for patient stratification in Vγ9Vδ2 T cell-based therapy

**Fig. 8:**
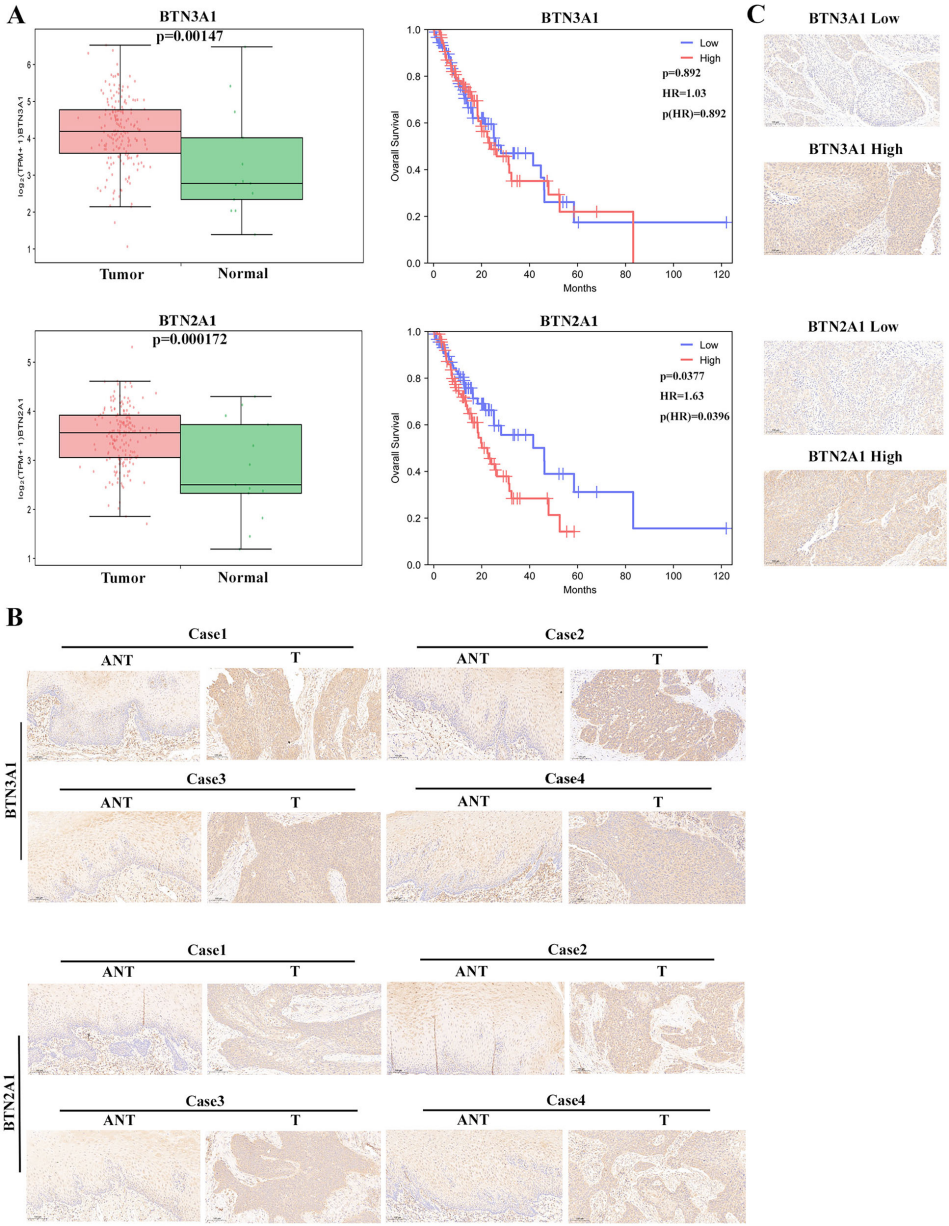
BTN3A1 and BTN2A1 expression profiles in tumor versus normal tissues and their association with Vγ9Vδ2 T cell-based immunotherapy. **A** Left panels: GEPIA3 database analysis showing the expression levels of BTN3A1 and BTN2A1 in tumor tissues (*n* = 182) versus normal tissues (*n* = 13). Right panels: Kaplan-Meier survival curves comparing overall survival between patients with low (*n* = 90) and high (*n* = 90) expression of BTN3A1 or BTN2A1. Statistical significance is indicated (*p* = 0.00147 for BTN3A1; *p* = 0.892 for BTN2A1, HR = 1.03, p(HR) = 0.892). **B** Representative immunohistochemical staining of BTN3A1 and BTN2A1 in paired adjacent normal tissues (ANT) and tumor tissues (T) from four ESCC patients (Case 1-4). **C** Representative images of low and high expression levels of BTN3A1 (up) and BTN2A1 (down) in ESCC tissues. Scale bars are indicated where applicable.

To determine whether BTN3A1 and BTN2A1 are detectable in clinical specimens of esophageal cancer, we first interrogated the GEPIA3 database. Both genes were significantly upregulated in tumor tissues compared with adjacent normal tissues (BTN3A1, *p* < 0.01; BTN2A1, *p* < 0.01; Fig. 8A). Survival analysis further demonstrated that high BTN2A1 expression was associated with poor overall survival (HR = 1.63, 95% CI 1.02–2.61, *p* < 0.05), whereas BTN3A1 expression showed no significant prognostic correlation (*p* > 0.05; Fig. 8A).

To corroborate these in silico observations at the protein level, we performed immunohistochemistry (IHC) on 10 paraffin‑embedded esophageal cancer specimens from our institutional cohort. Consistent with the transcriptomic data, both BTN3A1 and BTN2A1 were markedly elevated in tumor tissues relative to matched adjacent normal tissues (Fig. 8B). These findings confirm that both molecules are readily detectable in clinical samples and support their potential utility as tissue biomarkers for immune-based stratification.

Taken together, our results suggest that baseline expression levels of BTN3A1 and BTN2A1 may guide therapeutic stratification for Vγ9Vδ2 T cell‑based immunotherapy. Specifically, patients with low endogenous expression of these molecules might benefit from metformin combination therapy that upregulates BTN3A1/BTN2A1 and enhances γδ T cell recognition, whereas those with high expression may respond favorably to Vγ9Vδ2 T cell therapy alone (Fig. 8C). Nevertheless, these hypotheses warrant prospective validation in larger patient cohorts.

## Discussion

Metformin, a first-line medication for type 2 diabetes, has garnered significant interest in oncology due to emerging evidence suggesting potential antitumor effects mediated through metabolic pathway modulation and direct inhibition of cancer cell proliferation [34–37]. However, current evidence remains largely derived from preclinical studies and limited clinical observations, and it has not yet been established as a standard antineoplastic agent. Our study provides a comprehensive mechanistic elucidation of how metformin, beyond its classical applications, synergistically enhances the antitumor efficacy of Vγ9Vδ2 T cells against esophageal cancer. We systematically demonstrated that metformin acts as a potent modulator of the tumor-immune interface by significantly upregulating the expression of BTN3A1 and BTN2A1 on cancer cells. From an immunological perspective, this regulation may be mediated through the AMPK signaling pathway. Our preliminary data show that the AMPK inhibitor Compound C markedly suppresses metformin‑induced upregulation of both BTN3A1 and BTN2A1, suggesting that metformin acts, at least in part, via AMPK to control the expression of these butyrophilin molecules. Given that AMPK is a master metabolic sensor and can modulate multiple transcription factors (e.g., NLRC5, IRF1, or RUNX1), it is plausible that metformin‑activated AMPK directly or indirectly promotes BTN3A1/BTN2A1 transcription, thereby enhancing γδ T cell recognition and cytotoxicity. This finding positions metformin beyond its classical metabolic roles, revealing a novel immunomodulatory function that directly engages a key activation pathway for human γδ T cells. The observed upregulation was consistent across multiple cell lines and confirmed in vivo, establishing it as a robust phenomenon. This mechanism is particularly significant in the context of esophageal cancer, which often presents an immunosuppressive tumor microenvironment with limited endogenous T cell infiltration. By enhancing the expression of these critical phosphoantigen-presenting molecules, metformin effectively “primes” the tumor cells, making them more visible and susceptible to Vγ9Vδ2 T cell recognition. This bridges a critical gap in cancer immunotherapy, offering a strategy to enhance tumor immunogenicity against solid malignancies that are traditionally resistant to immune attack.

The functional consequences of this molecular priming were profound and multi-faceted. The in vitro cytotoxicity assays demonstrated clear synergy, which was quantitatively confirmed by combination index analysis. This was not merely a result of enhanced ligand expression but was coupled with a significant improvement in Vγ9Vδ2 T cell functionality, as evidenced by increased CD69 activation, elevated CD107a degranulation, and robust production of IFN-γ and TNF-α. Crucially, our blocking experiments with an anti-BTN3A1 antibody provided direct causal evidence, as the antibody specifically reversed the metformin-mediated enhancement of cytotoxicity, returning cytotoxicity to the level observed with Vγ9Vδ2 T cells alone. This confirms that the BTN2A1-BTN3A1 axis is the indispensable functional bridge for the observed synergy. The translational relevance of these findings was strongly supported by our in vivo data, where the combination therapy achieved superior tumor control in two independent xenograft models. The sustained suppression of tumor growth, coupled with the lack of significant toxicity, underscores the therapeutic potential of this approach.

Beyond the direct cytotoxic synergy between metformin and Vγ9Vδ2 T cells, metformin may also orchestrate broader immunomodulatory changes within the esophageal carcinoma tumor microenvironment (TME) that further amplify antitumor immunity. Accumulating evidence indicates that metformin can remodel the immune landscape of colorectal cancer, influencing not only CD8⁺ T cells but also B cells, macrophages, dendritic cells, and myeloid-derived suppressor cells (MDSCs) in a context-dependent manner [38, 39]. More specifically, metformin suppresses the immunosuppressive function of MDSCs by downregulating CD39 and CD73 expression and their ectoenzymatic activity, thereby reducing adenosine-mediated immune suppression and restoring T cell effector functions [40]. Regarding tumor-associated macrophages (TAMs), metformin promotes the repolarization of pro-tumoral M2-like macrophages toward an anti-tumoral M1 phenotype, shifting the TME from an immunosuppressive to a pro-inflammatory state [41]. Additionally, metformin has been reported to reduce the infiltration of regulatory T cells (Tregs) in certain tumor models, further alleviating immunosuppressive networks. Collectively, these direct effects on multiple immune lineages suggest that metformin not only enhances Vγ9Vδ2 T cell functionality but also reshapes the TME into a more permissive environment for sustained T cell infiltration, activation, and tumor control. Thus, the superior antitumor efficacy observed in our combination regimen likely stems from a dual-layer mechanism: metformin simultaneously boosts the intrinsic cytotoxic capacity of Vγ9Vδ2 T cells while alleviating multiple layers of immunosuppression within the TME, establishing a coordinated immunotherapeutic attack that may be more durable and resistant to adaptive resistance mechanisms [42].

The clinical implications of our work are substantial. Vγ9Vδ2 T cell-based therapies have faced challenges, including variable patient responses and limited infiltration into solid tumors [36, 37]. Our findings suggest that pre-treatment or co-administration with metformin could represent a straightforward and cost-effective strategy to overcome these limitations. Metformin’s excellent safety profile and low cost make it an ideal candidate for combination regimens [43–46]. Future clinical trials should explore the sequencing and dosing of metformin with adoptive Vγ9Vδ2 T cell transfer in patients with esophageal carcinoma. Furthermore, it would be highly relevant to investigate whether metformin can similarly sensitize other solid tumor types to γδ T cell therapy, potentially expanding its application across oncology. Another promising direction lies in combining this dual therapy with immune checkpoint inhibitors. It is plausible that metformin-induced T cell activation and infiltration, as demonstrated by our Granzyme B and TUNEL assay data, could help reverse T cell exhaustion and synergize with PD-1/PD-L1 blockade, creating a multi-pronged immunotherapeutic attack.

**Fig. 9:**
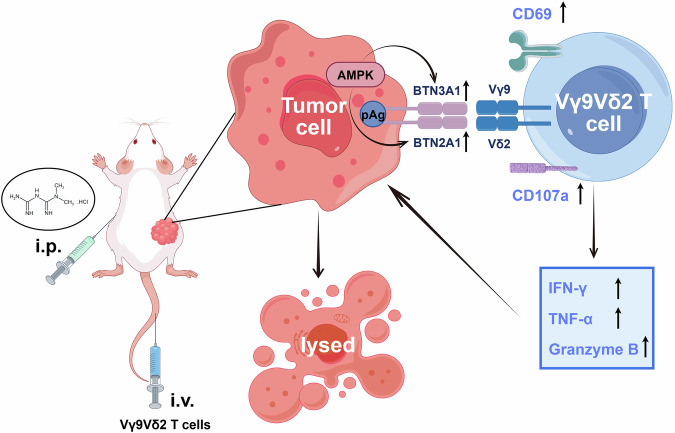
The working model illustrating the role of BTN3A1/BTN2A1 upregulation by metformin enhances Vγ9Vδ2 T cell killing.

In conclusion, our research delineates a complete mechanistic pathway from metformin-induced upregulation of BTN3A1/BTN2A1 on tumor cells to enhanced Vγ9Vδ2 T cell activation, and cytotoxic function, culminating in potent and well-tolerated antitumor activity (Fig. 9). We have transformed the understanding of metformin from a metabolic drug to a credible immunomodulator that can effectively condition the tumor microenvironment for superior γδ T cell recognition. This work provides a strong preclinical rationale for repurposing metformin as an adjunct in cancer immunotherapy. The combination of metformin and Vγ9Vδ2 T cells represents a promising, readily translatable strategy that could potentially improve outcomes for patients with esophageal cancer and possibly other solid malignancies, heralding a new avenue for enhancing cell-based immunotherapies.

## Supplementary information

uncropped Blots

Supplementary DATA

## Data Availability

The datasets used and/or analysed during the current study are available from the corresponding author on reasonable request.
