# Supplementary material for: Metformin sensitizes esophageal squamous cell carcinoma to Vγ9Vδ2 T cell-mediated cytotoxicity by upregulating BTN3A1 and BTN2A1
Source: Cell Death Dis. 2026 Jun 6;17(1):708. doi: 10.1038/s41419-026-08924-6 (PMC13458587; doi:10.1038/s41419-026-08924-6)

Figure 1

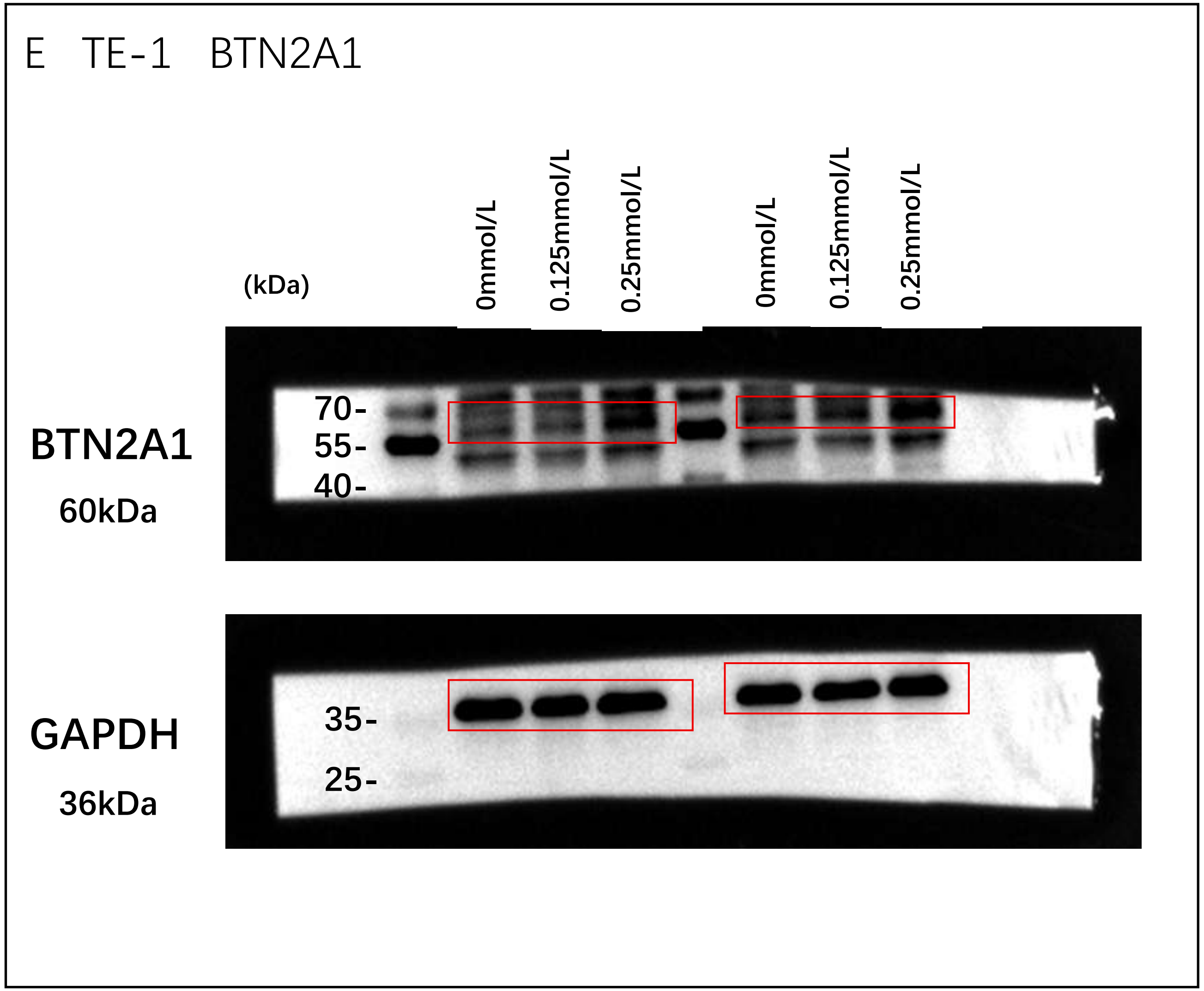
E TE-1 BTN2A1
(kDa)
0mmol/L
0.125mmol/L
0.25mmol/L
0mmol/L
0.125mmol/L
0.25mmol/L
BTN2A1
60kDa
70-
55-
40-
GAPDH
36kDa
35-
25-

Figure 1

E TE-1 BTN3A1

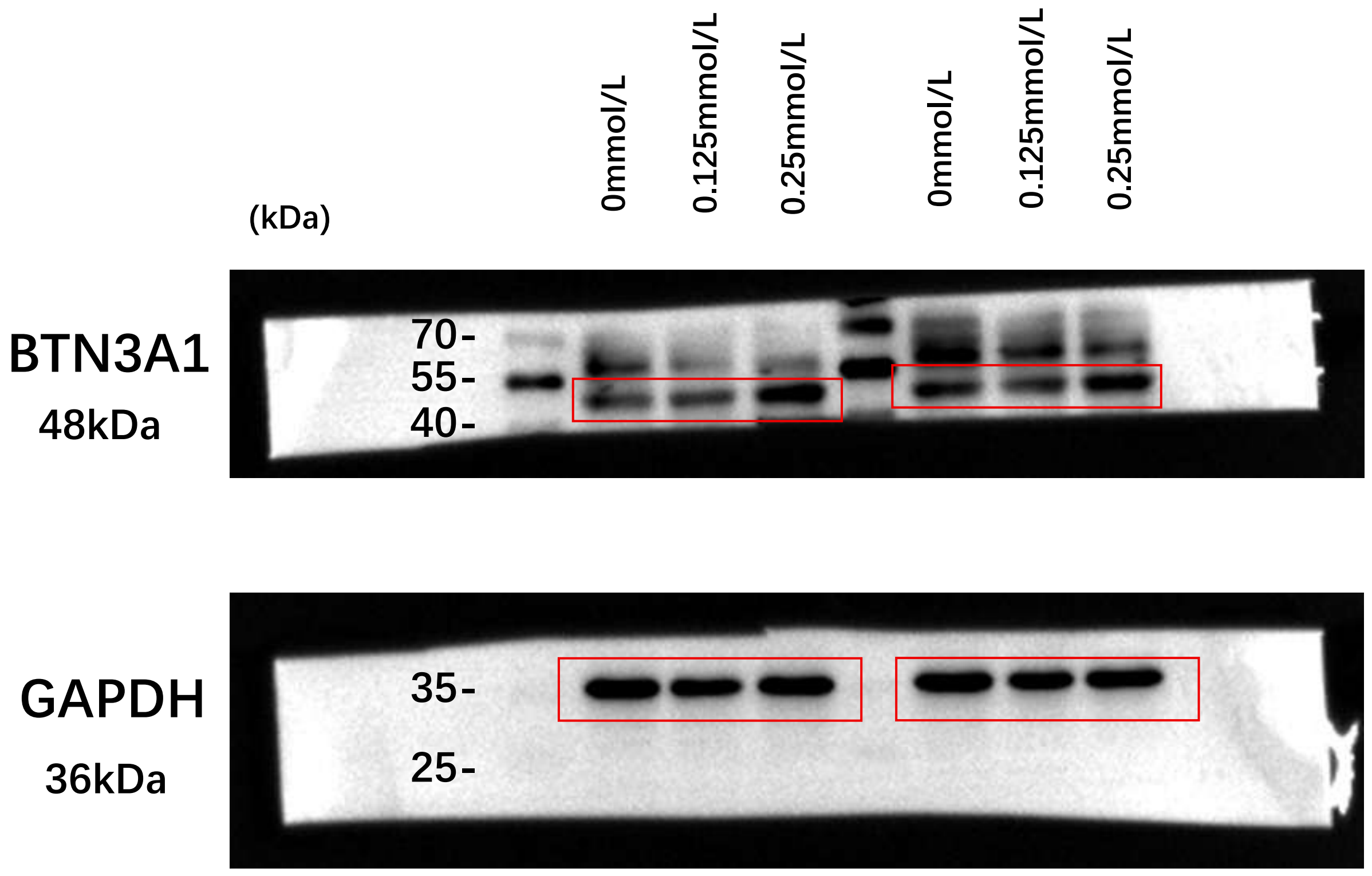

Figure 1

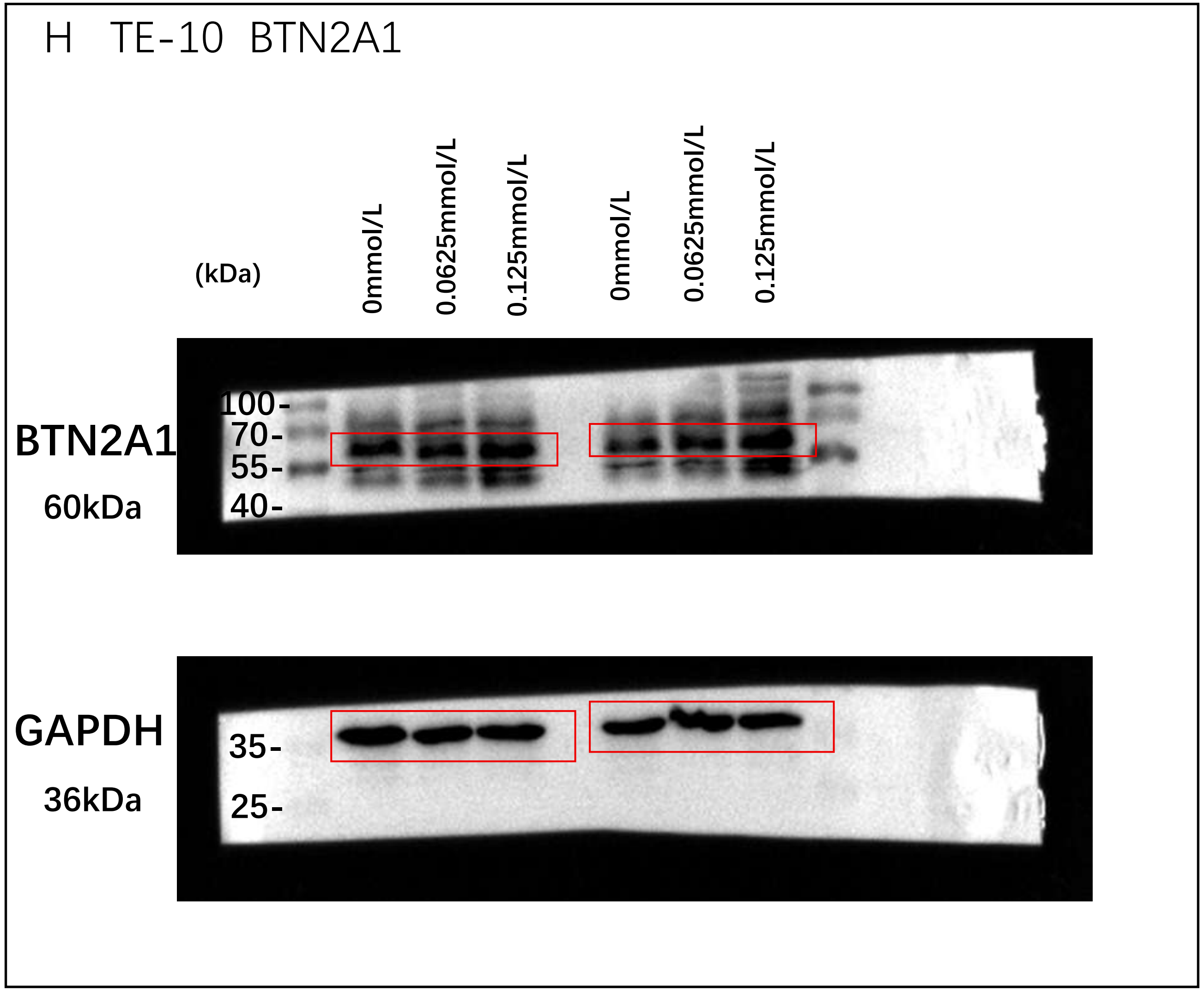

H TE-10 BTN2A1
(kDa)
0mmol/L
0.0625mmol/L
0.125mmol/L
0mmol/L
0.0625mmol/L
0.125mmol/L
BTN2A1
60kDa
100-
70-
55-
40-
GAPDH
36kDa
35-
25-

Figure 1

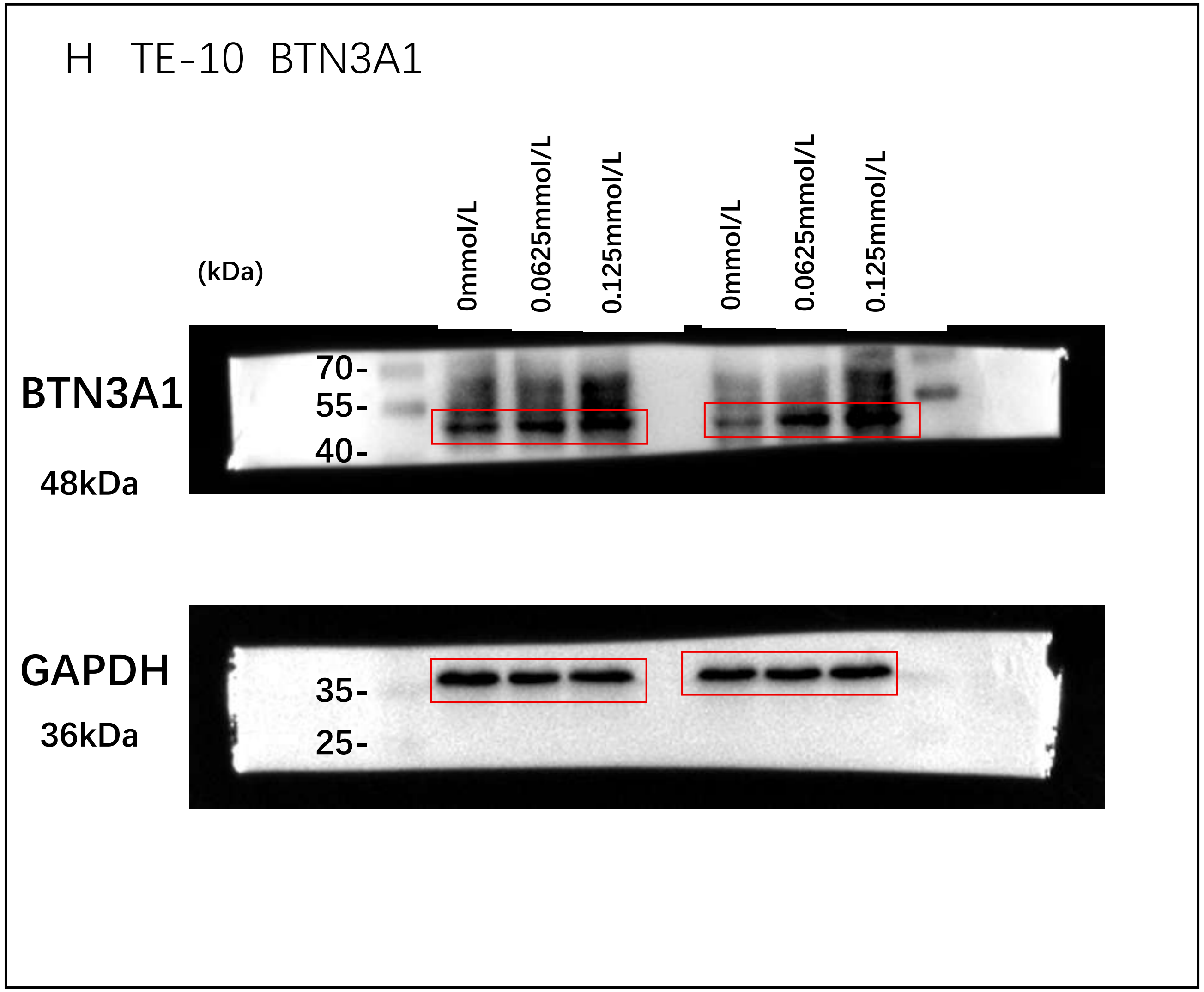
H TE-10 BTN3A1
(kDa)
0mmol/L
0.0625mmol/L
0.125mmol/L
0mmol/L
0.0625mmol/L
0.125mmol/L
BTN3A1
48kDa
70-
55-
40-
GAPDH
36kDa
35-
25-

Supplementary Figure 1

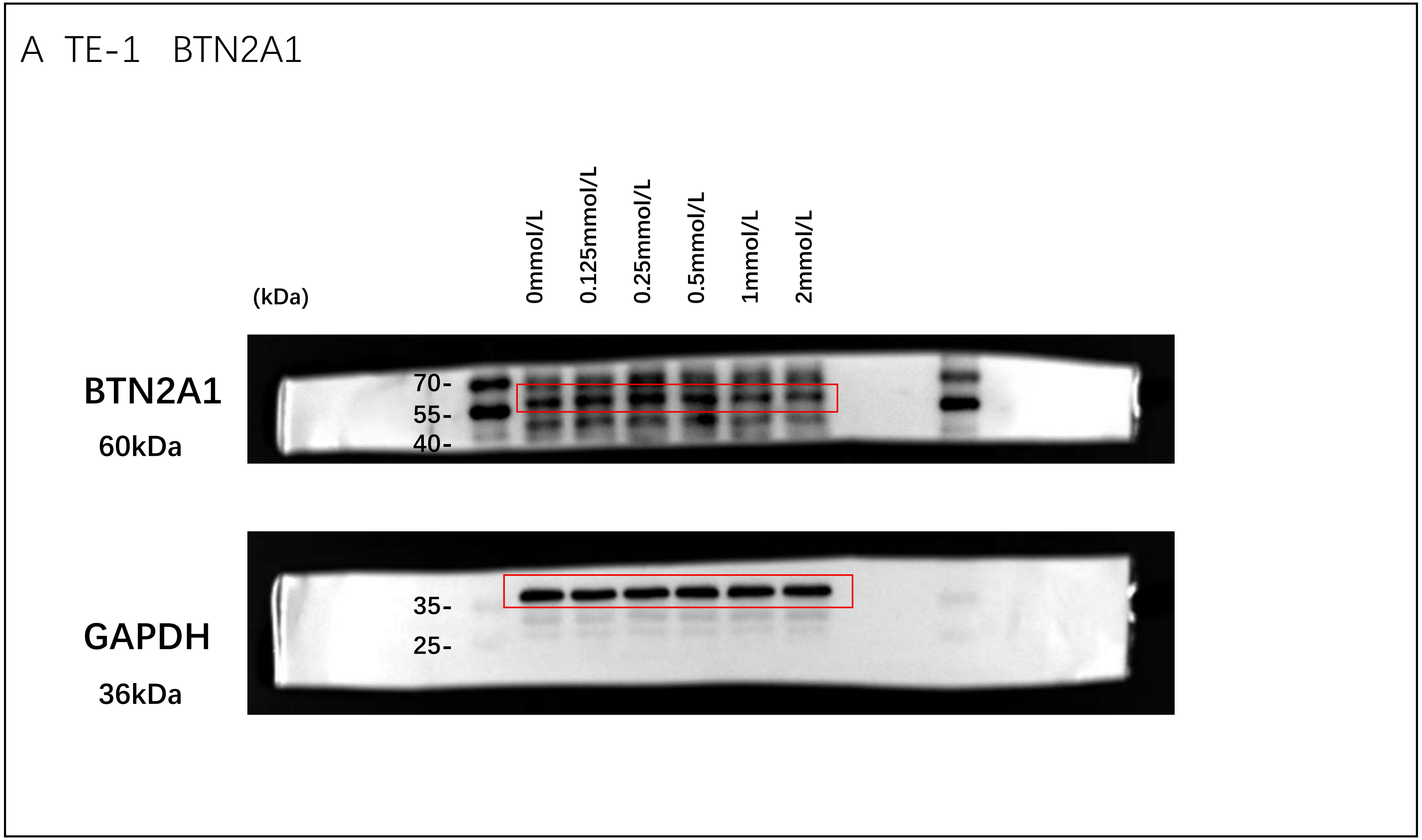

Supplementary Figure 1

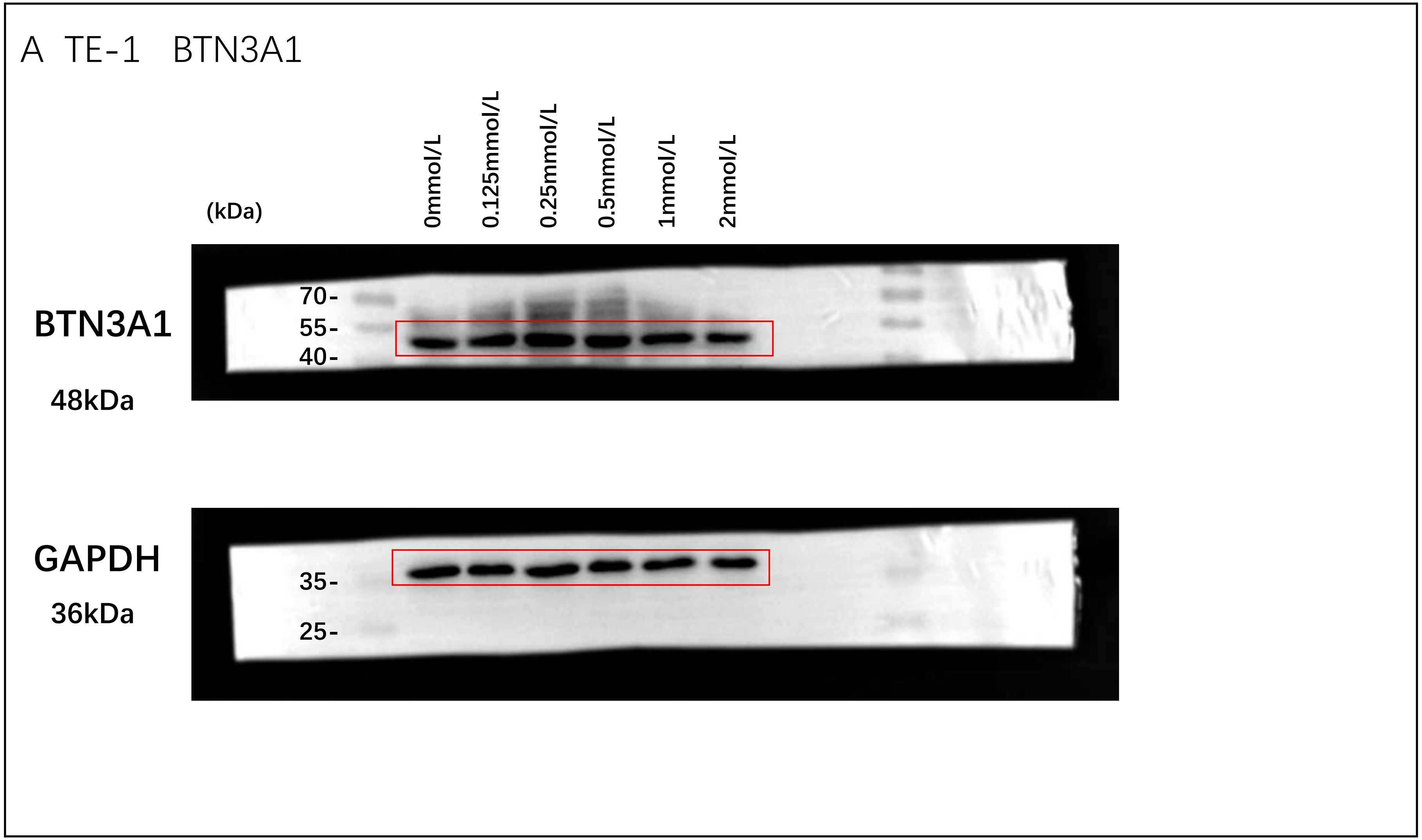

Supplementary Figure 1

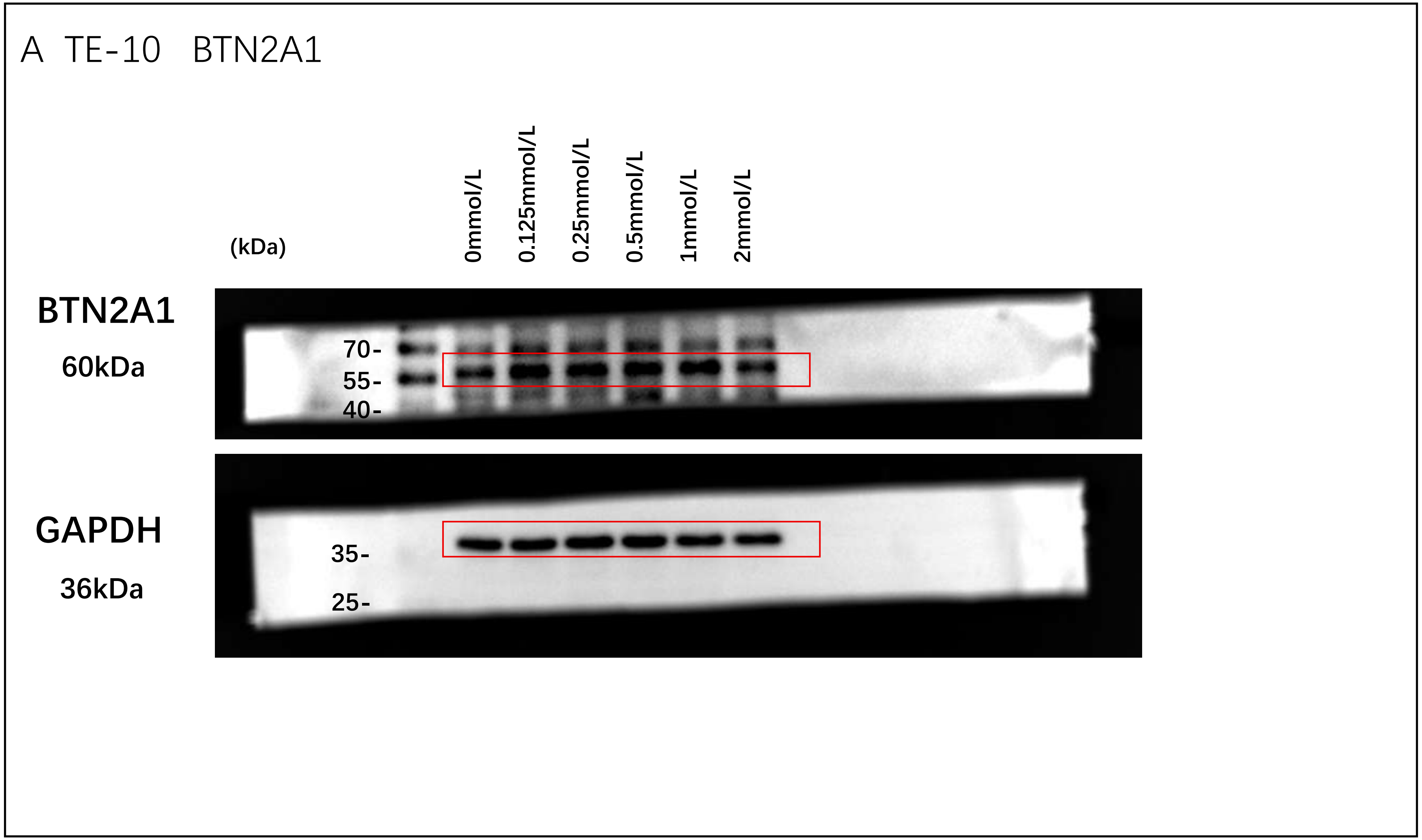

Supplementary Figure 1

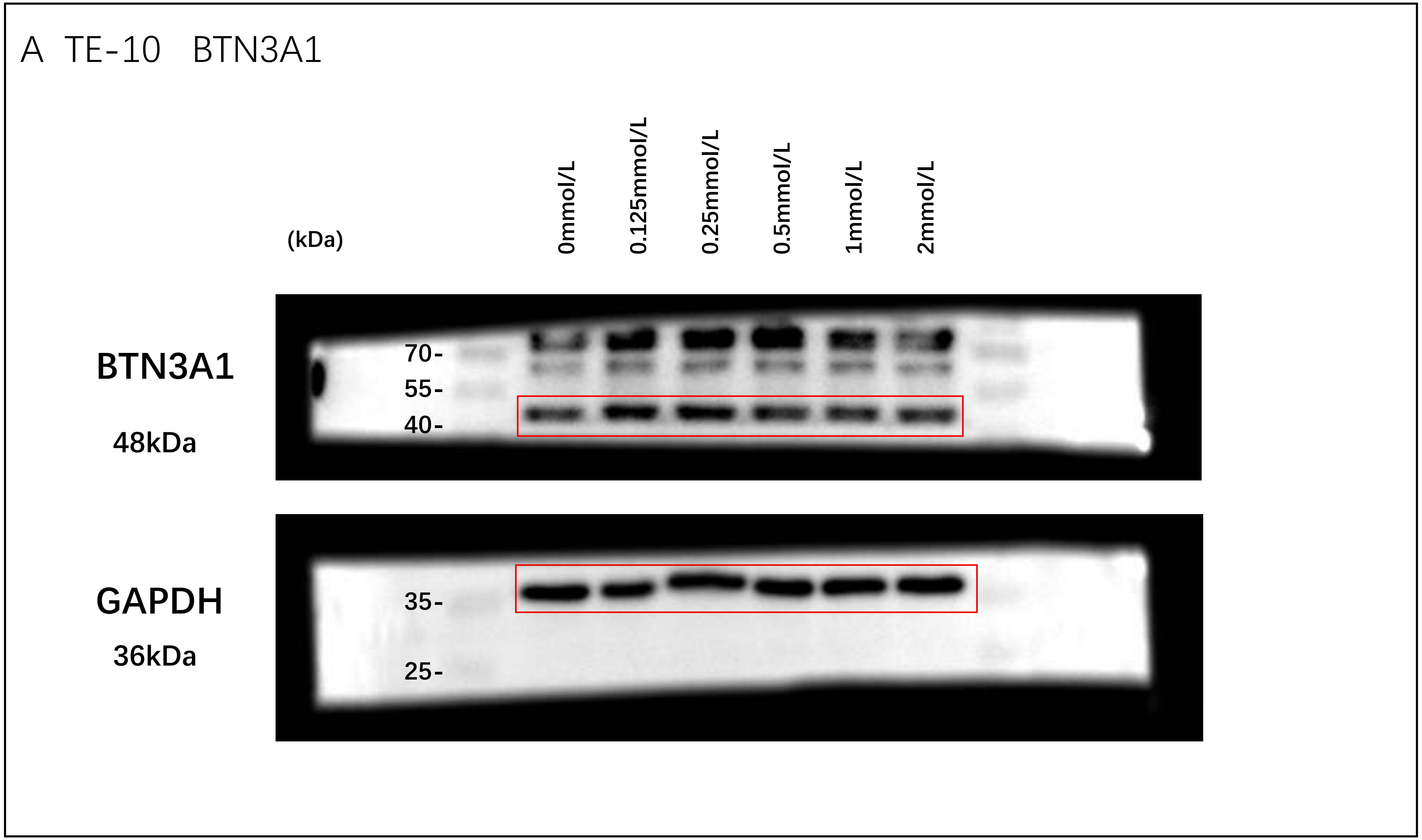

Supplementary Fig 3

TE-1 BTN2A1

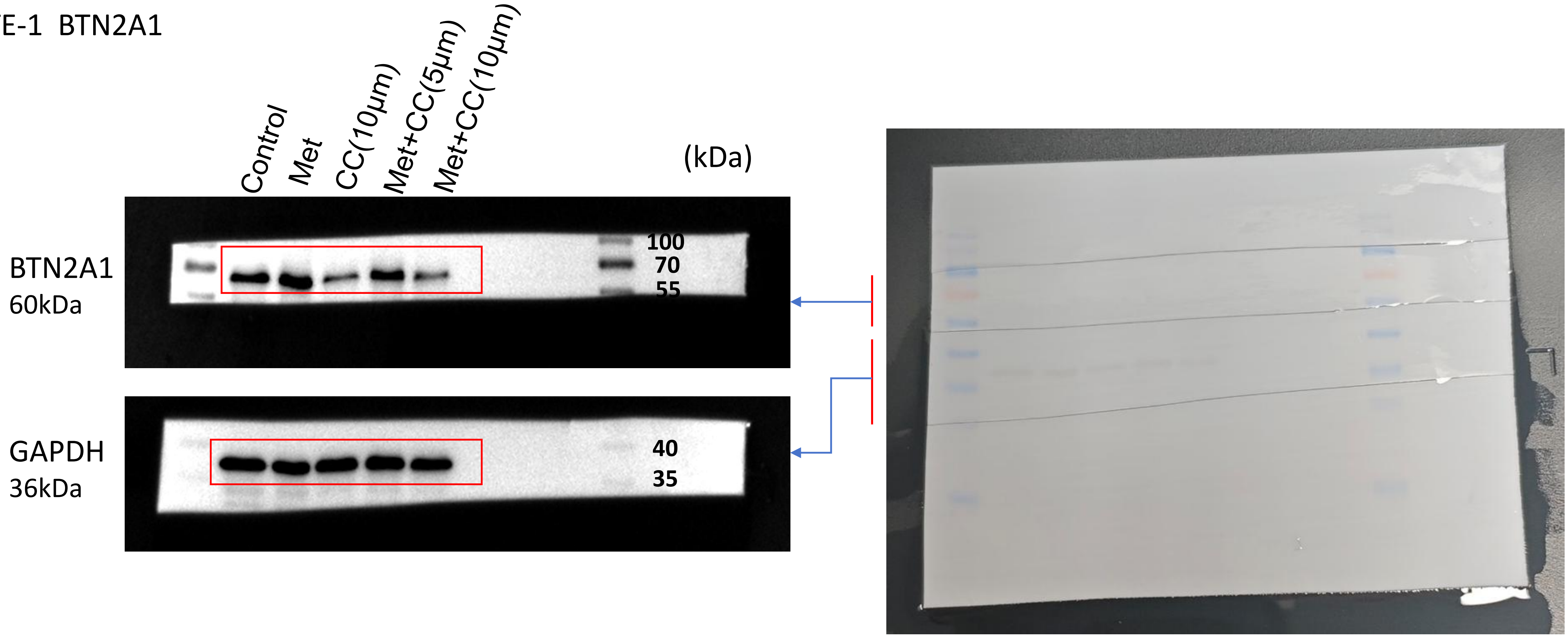

Supplementary Fig 3

TE-10 BTN2A1

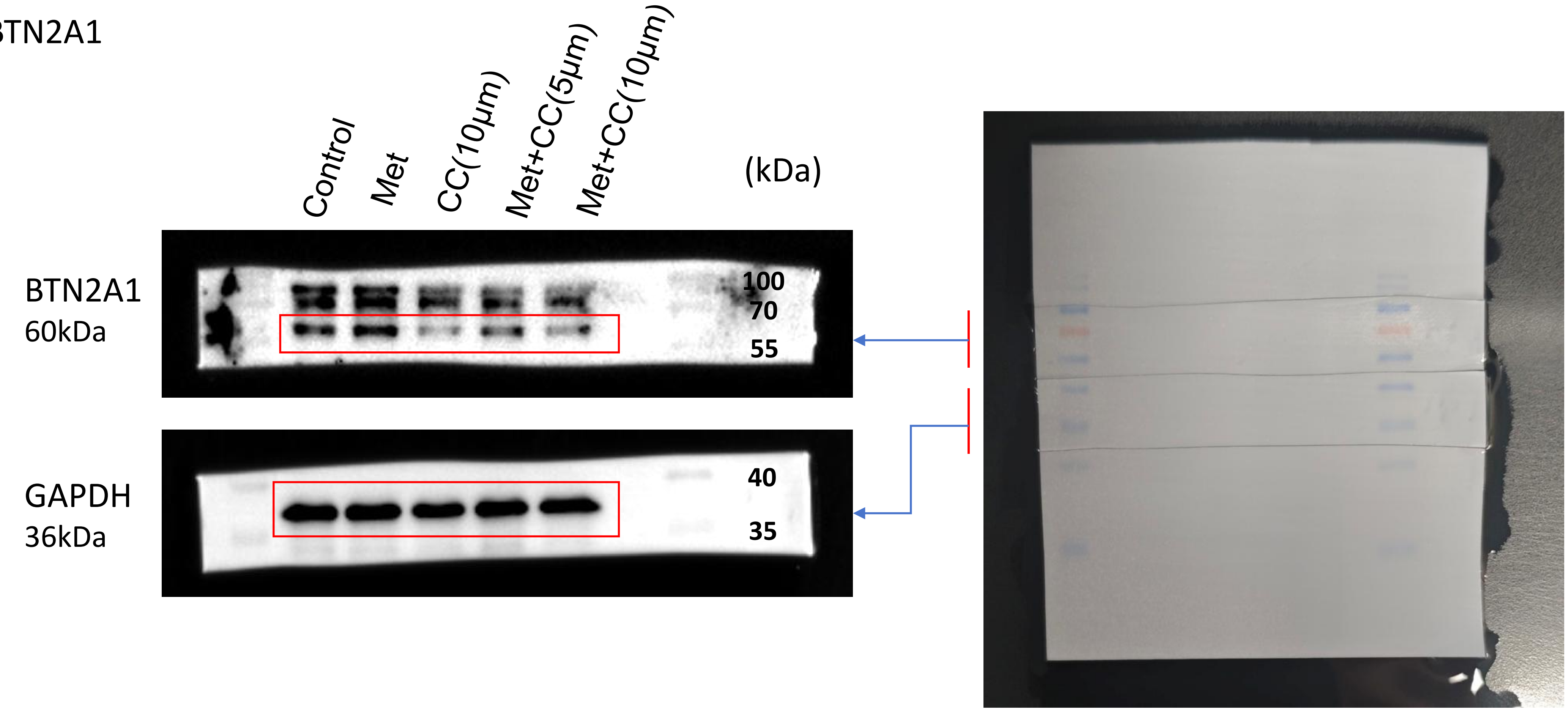

Supplementary Fig 3

TE-1 BTN3A1

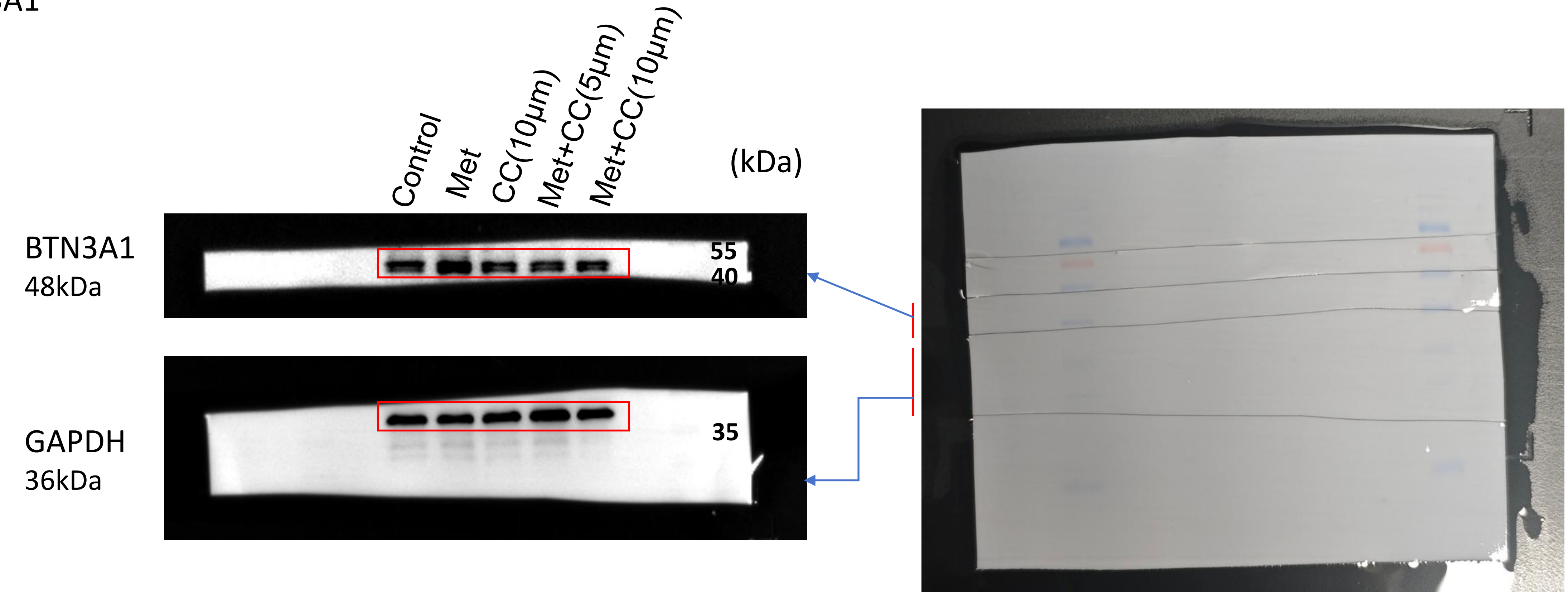

Supplementary Fig 3

TE-10 BTN3A1

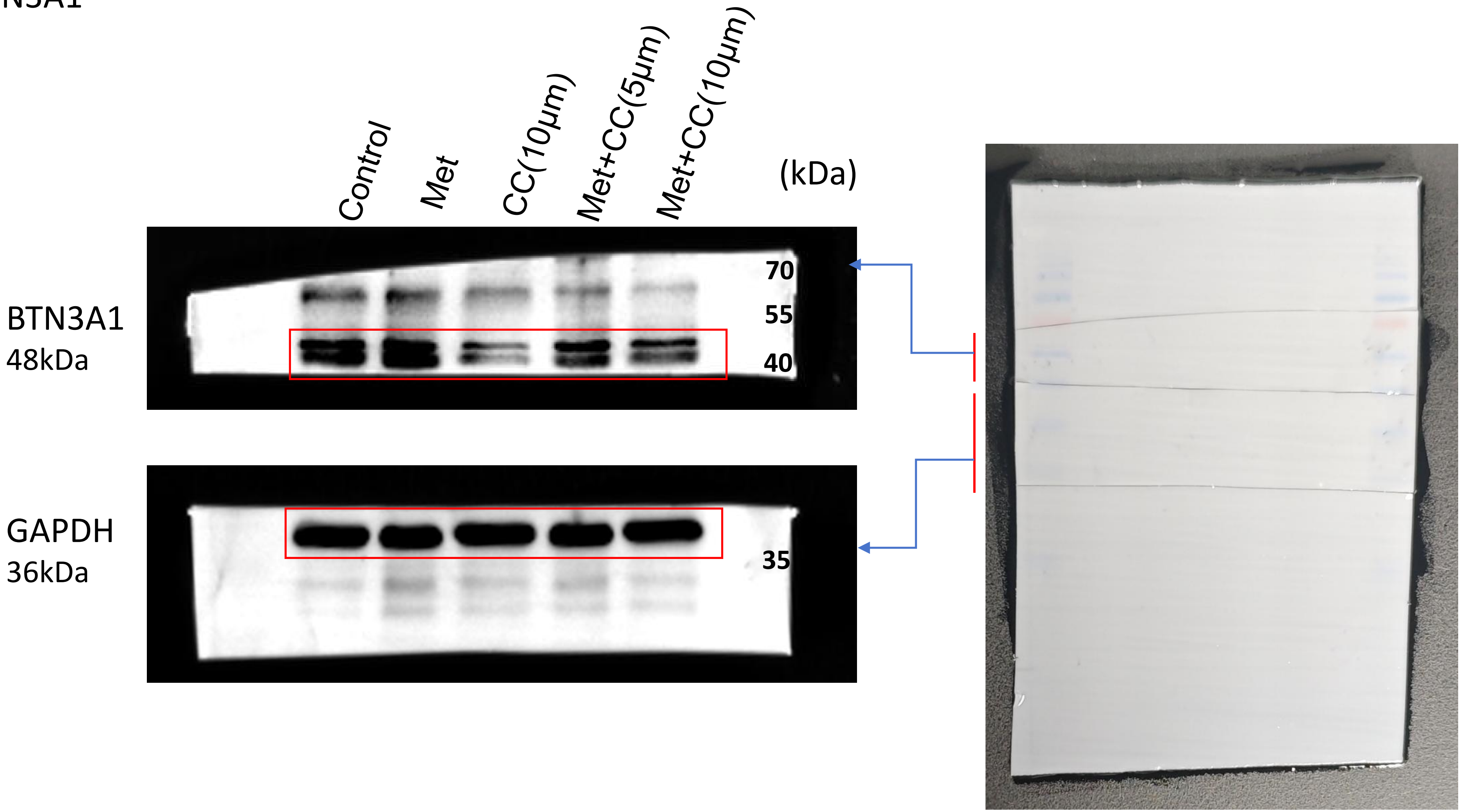

Supplement: Supplementary file 1 [file 41419_2026_8924_MOESM1_ESM.pdf]
