# Supplementary material for: Metformin sensitizes esophageal squamous cell carcinoma to Vγ9Vδ2 T cell-mediated cytotoxicity by upregulating BTN3A1 and BTN2A1
Source: Cell Death Dis. 2026 Jun 6;17(1):708. doi: 10.1038/s41419-026-08924-6 (PMC13458587; doi:10.1038/s41419-026-08924-6)
Supplement: Supplementary file 2 [file 41419_2026_8924_MOESM2_ESM.docx]

**Supplementary Fig. 1 Metformin upregulates BTN2A1 and BTN3A1 expression in esophageal cancer cells under different concentration conditions.**

(A) Western blot analysis of BTN2A1 and BTN3A1 protein expression in TE-1 and TE-10 cells treated with the indicated concentrations of metformin (0-2 mM) for 24 hours. GAPDH was used as a loading control.

(B, C) Densitometric quantification of BTN2A1 (B) and BTN3A1 (C) protein levels in TE-1 cells from (A), normalized to GAPDH. Data are presented as mean ± SD (n=3 independent experiments). Statistical analysis was performed using one-way ANOVA with Dunnett's post-hoc test versus the 0 mM control group. *p < 0.05.

(D, E) Densitometric quantification of BTN2A1 (D) and BTN3A1 (E) protein levels in TE-10 cells from (A), normalized to GAPDH. Data are presented as mean ± SD (n=3 independent experiments). Statistical analysis was performed using one-way ANOVA with Dunnett's post-hoc test versus the 0 mM control group. *p < 0.05, **p < 0.01.

**Supplementary Fig. 2** **Effects of metformin treatment on the expression of BTN3A2 and BTN3A3 in esophageal squamous cell carcinoma cell lines.**

(A-B) Quantitative real-time PCR (qPCR) analysis of BTN3A2 expression in TE-1 and TE-10 cells treated with metformin for the indicated concentrations of metformin. Relative expression levels were normalized to untreated controls. Data are representative of three independent experiments (ns, p > 0.05).

(C-D) qPCR analysis of BTN3A3 expression in TE-1 and TE-10 cells following metformin treatment. Statistical significance is indicated where applicable (ns: not significant; **p < 0.01). Data are presented as mean ± SD from at least three replicates.

**Supplementary Fig. 3 Metformin upregulates BTN3A1 and BTN2A1 expression in an AMPK-dependent manner.**

(A) Representative Western blot analysis of BTN3A1 and BTN2A1 protein levels in TE1 cells treated with control, metformin (Met), Compound C (CC, an AMPK inhibitor) at 10 μM, or a combination of metformin and CC at indicated concentrations (5 μM or 10 μM). GAPDH served as a loading control. (B) Quantification of relative protein expression levels of BTN3A1 and BTN2A1 normalized to GAPDH in TE1 cells (data are shown as mean ± SD, *p < 0.05, **p < 0.01, ***p < 0.001). (C) Western blot analysis of BTN3A1 and BTN2A1 expression in TE-10 cells under the same treatment conditions as in (A). (D) Quantification of relative protein expression levels in TE-10 cells normalized to GAPDH.

**Supplementary Fig. 4 Metformin does not directly modulate γδ T cell effector functions in the absence of tumor cells.**

Human Vγ9Vδ2 T cells were isolated and pretreated with metformin (0.25 mM ) in the absence of tumor cells. Subsequently, cells were assessed for (A) proliferation (Ki67 expression), (B) activation (CD69 expression), (C) degranulation (CD107a surface mobilization), and (D-E) cytokine production (intracellular IFN‑γ and TNF‑α). All experiments were performed at least three times with similar results. Statistical significance was determined using t-tests ( ns, not significant).

**Supplementary Fig. 5** **Assessment of renal and hepatic toxicity following combination treatment with metformin and Vγ9Vδ2 T cells.**

Organ were harvested at the endpoint of the efficacy study involving treatment with metformin, Vγ9Vδ2 T cells, or their combination. Histopathological examination reveals no evidence of significant morphological damage in either organ across all treatment groups compared to controls, indicating a lack of overt treatment-related toxicity at the administered doses.
